# Disproportionate burden of violence: Explaining racial and ethnic disparities in potential years of life lost among homicide victims, suicide decedents, and homicide-suicide perpetrators

**DOI:** 10.1371/journal.pone.0297346

**Published:** 2024-02-07

**Authors:** Gregory M. Zimmerman, Emma E. Fridel, Daniel Trovato

**Affiliations:** 1 School of Criminology and Criminal Justice, Northeastern University, Boston, MA, United States of America; 2 College of Criminology and Criminal Justice, Florida State University, Tallahassee, FL, United States of America; Sapienza, University of Rome, ITALY

## Abstract

Research indicates that the burden of violent death in the United States is disproportionate across racial and ethnic groups. Yet documented disparities in rates of violent death do not capture the full extent of this inequity. Recent studies examining race-specific rates of potential years of life lost—a summary measure of premature mortality—indicate that persons of color may die at younger ages than their counterparts, leading to increased trauma among surviving family members, friends, and communities. This study examines racial and ethnic disparities in potential years of life lost among people who died by homicide and suicide. We calculated potential years of life lost using life expectancy values specific to each racial and ethnic group, thereby isolating racial differences in potential years of life lost due to violence. Findings indicated that persons of color were disproportionately impacted by violence. Non-Hispanic African American homicide victims, suicide decedents, and homicide-suicide perpetrators died eleven or more years earlier than their non-Hispanic White counterparts. Similar disparities were observed for non-Hispanic Asian or Pacific Islander decedents. Less pronounced differences were observed for Hispanic and non-Hispanic American Indian or Alaska Native decedents. These racial and ethnic disparities were partly accounted for by a broad array of individual differences, incident characteristics, and contextual factors. The results suggest that homicide and suicide exact a high societal cost, and the burden of that cost is disproportionately high among persons of color.

## Introduction

Lethal violence represents a public health crisis in the United States. In 2020, approximately 46,000 persons died by suicide (14.0 deaths per 100,000 population) and more than 25,000 persons died by homicide [1]. The one-year increase in homicide from 6.0 deaths per 100,000 in 2019 to 7.8 deaths per 100,000 in 2020 represented the largest single-year increase in over a century [1]. Additionally, homicide-suicide accounts for 5% of U.S. homicides annually, or approximately 1,000 to 1,500 deaths [2]. Beyond loss of life, lethal violence has wide-reaching impacts on victims’ family members, friends, and broader communities. Psychological effects include depression, anxiety, anger, and guilt. Socio-behavioral effects include school-related and work-related problems, suicidal ideation, and aggression [3]. Violent deaths also pose a significant financial burden for survivors, employers, and taxpayers, including funeral costs and medical bills, investments in the criminal justice system, lost wages and productivity, devaluation of property, and other quality of life losses [4]. The Centers for Disease Control and Prevention [5] estimated the economic costs of homicides and suicides, including medical costs and value of statistical life, as approximately $670 billion in the U.S. in 2019 [6]. These consequences may be amplified for homicide-suicide events, which involve multiple deaths.

The social burden of lethal violence, however, is not born equally by all Americans. Scholars have consistently documented disparities in rates of lethal violence across racial and ethnic groups. The most recent race-specific age-adjusted homicide rates are 33.6 per 100,000 for African American persons, 12.9 for American Indian and Alaska Native persons, 6.9 per 100,000 for Hispanic persons, 3.3 per 100,000 for White persons, and 1.7 for Asian and Pacific Islander persons. Suicide rates are highest for American Indian and Alaska Native (28.1 per 100,000) and White (17.4 per 100,000) persons, followed by African American (8.7 per 100,000), Hispanic (7.9 per 100,000), and Asian and Pacific Islander (6.8 per 100,000) persons [1]. *Rates* of violent deaths do not tell the full story, however, as they fail to account for the age at which a person dies.

One way to compare the burden of premature violent death across racial and ethnic groups is by measuring potential years of life lost [7]—the number of years a person would have lived had they survived [8]. As an alternative to death rates, potential years of life lost gives more weight to deaths occurring among younger persons. Recent research using a measure of potential years of life lost indicates that persons of color may be doubly victimized by homicide: they are more likely to be victims of homicide *and* to be victimized at younger ages [9]. Similarly, suicide among all non-White racial and ethnic groups increased from 2018 to 2021 [10], while suicide among the non-Hispanic White population decreased [11], suggesting that persons of color may be disproportionately burdened by suicide.

Despite these implications, few studies have examined the mechanisms responsible for racial and ethnic disparities in age at time of violent death [12, 13]. Accordingly, this study uses data on homicide, suicide, and homicide-suicide incidents from the National Violent Death Reporting System (NVDRS) appended to contextual information on census-designated places from the American Community Survey (ACS) to examine: the correlates of potential years of life lost among homicide victims, suicide decedents, and homicide-suicide perpetrators in the U.S. from 2003 to 2019; and the extent to which racial and ethnic disparities in potential years of life lost among persons who experienced violent deaths can be accounted for by a constellation of individual, incident, socio-familial, and contextual characteristics. We begin with a brief overview on racial and ethnic disparities in violent death and age at time of death before discussing the factors that may account for them.

### Racial and ethnic disparities in violent deaths

Racial and ethnic disparities among homicide victims, suicide decedents, and homicide-suicide perpetrators are well established in the extant literature. In his seminal study on violence in Philadelphia, Wolfgang (1958, p. 31) [14] cited the overrepresentation of persons of color among homicide victims as “an incontrovertible fact.” These racial and ethnic disparities in homicide victimization have persisted through the crime rate drop in the 1990s [15], the September 11^th^ terrorist attack and its aftermath in the 2000s [16, 17], and the emergence of the Black Lives Matter movement and the push toward criminal justice reform in the 2010s [18]. The most recent statistics indicate that homicide rates are almost ten, four, and two times higher among non-Hispanic African American, non-Hispanic American Indian and Alaska Native, and Hispanic persons, respectively, than among their White counterparts [1].

There is also wide variation in suicide rates across racial and ethnic groups. The suicide rate is consistently higher among non-Hispanic American Indian and Alaska Native persons and non-Hispanic White persons than among Hispanic, non-Hispanic African American, and non-Hispanic Asian and Pacific Islander persons [1]. Yet, there is evidence that suicide rates have recently been decreasing for White persons but increasing among the non-White population. Between 2018 and 2021, the age-adjusted suicide rate decreased by 4.5% among non-Hispanic White persons but increased among all non-White racial and ethnic groups [10, 11].

Homicide-suicide tends to be a predominantly White phenomenon. Research has indicated that White homicide offenders are at least two times more likely than non-White homicide offenders to commit suicide [17, 19, 20]. Additionally, persons who die by suicide following homicide are more likely than homicide offenders, but less likely than persons who die by suicide, to be White [21].

### Racial and ethnic disparities in age at time of violent death

Moving beyond overall violent death rates, recent research provides evidence of significant racial and ethnic disparities in age at time of violent death, with a disproportionate burden of homicide victimization and suicide among younger persons of color. For example, in 2020, homicide was the fourth leading cause of death among non-Hispanic White persons aged 1 to 9 years and the fifth most common cause of death among White persons aged 10–34 years. But, for African American residents, homicide was the second leading cause of death among persons aged 1 to 14 years and the single most common cause of death among persons aged 15–24 years. For American Indian and Alaska Native residents, homicide was the second leading cause of death among persons aged 1 to 9 years and the third most common cause of death for persons aged 15 to 24 years. For Hispanic residents, homicide was the fourth leading cause of death among persons aged 1 to 14 years and the third most common cause of death among persons aged 15–34 years. Homicide was approximately the fourth leading cause of death among all Asian or Pacific Islander age groups under 34 years of age [1]. Accordingly, research has demonstrated that the association between age and homicide victimization is stronger among the non-White population than the White population [22, 23]. Persons of color are thus more often victims of homicide *and* victimized at younger ages, compared to their White counterparts.

Concerning suicide, much has been made of the two consecutive year decline in suicide from 2018 to 2020 [11], but disaggregating changes in suicide by race and ethnicity and age reveal interesting differences. In fact, the only racial and ethnic group to experience an age-adjusted decrease in suicide from 2018 to 2021 was non-Hispanic White persons, from 18.1 per 100,000 persons to 17.4, a 3.9% decrease. Age-adjusted suicide rates increased for every other racial and ethnic group. For non-Hispanic American Indian or Alaska Native persons, the age-adjusted suicide rate increased 26.0% from 22.3 in 2018 to 28.1 in 2021. For non-Hispanic African American persons and Hispanic persons, the age-adjusted suicide rate increased 19.2% and 6.8%, respectively. The increase in suicide among Asian and Pacific Islander persons was more modest. These increases were particularly apparent among younger age groups. The suicide rate among non-Hispanic African American, non-Hispanic American Indian or Alaska Native, and Hispanic persons aged 10 to 24 years increased 36.6%, 16.7%, and 8.2%, respectively, from 2018 to 2021 [10]. Thus, non-White persons may not be as likely to die by suicide overall, but disaggregating by age and race demonstrates a disproportionate burden of suicide among younger persons of color.

To more completely assess racial and ethnic disparities in the social burden of homicide victimization and suicide, Rosenberg and colleagues (2017) [8] measured potential years of life across race and ethnicity for the top 39 causes of death in the U.S. in 2015. They demonstrated that Black Americans had more potential years of life lost per death than White Americans for each of the 39 top causes of death. The causes of death that resulted in the largest racial differences in age at the time of death were congenital and chromosomal abnormalities, followed by suicide and homicide (which resulted in a greater absolute number of deaths and potential years of life lost than congenital and chromosomal abnormalities). “The average black American committing suicide was 10 years younger than the average white American (38 years old versus 48 years old). Homicide, the leading contributor to PYLL among black Americans, had the third highest racial disparity in average age at death (30 years old in black Americans versus 37 years old in white Americans)” ([8], p. 4). Similarly, examining 6,000 intimate partner homicide victims from the National Violent Death Reporting System (2006–2015), Graham and colleagues (2021 [9]), found that American Indian and Alaska Native, African American, and Hispanic intimate partner homicide victims died more than nine years earlier than their White counterparts. Similarly, Asian and Pacific Islander intimate partner homicide victims died approximately five years earlier than their White counterparts.

#### Explaining racial and ethnic disparities in age at time of violent death

While racial and ethnic disparities in age at time of violent death are well-documented, their etiology remains a mystery. This is particularly concerning, for as Rosenberg et al. ([8], p. 1–2) argue, the lack of research and public resource investment in understanding these differences threatens to perpetuate the disproportionate disadvantage on persons of color. Acknowledging critiques that race and ethnicity do not hold etiologic credibility as causes of violence [24, 25], we instead investigate whether differential exposure to violence-inducing conditions among persons of color impact racial and ethnic disparities in potential years of life lost due to violence. As discussed below, prior research suggests that persons of color are more likely to experience individual, incident, socio-familial, and contextual risk factors for homicide and suicide. In order to isolate the effects of race and ethnicity on potential years of life lost due to violence, empirical models must account for the risk factors for violence that are disproportionately experienced by persons of color. Accounting for these risk factors should at least partly reduce observed racial and ethnic gaps in potential years of life lost due to violence. The empirical goal of the manuscript is to establish racial and ethnic differences in potential years of life lost due to violence, net of a broad array of risk factors for homicide and suicide that are differentially experienced across racial and ethnic groups. We discuss three groups of risk factors for violence grounded in prior research on race and ethnicity.

One perspective focuses on racial and ethnic disparities in a variety of individual characteristics, including socioeconomic status, occupational prestige, and educational attainment. Unnever and Gabbidon (2011 [26]), for example, contend that persons of color have lower levels of socioeconomic status, employment success, and education due to structural restraints and systemic injustice [27, 28]. Similarly, persons of color may be more vulnerable to the detrimental consequences of alcohol use [29, 30], drug use such as opioids [31], and substance use more broadly [32]. Although never tested empirically, the implication is that accounting for these factors in statistical models may reduce racial and ethnic differences in potential years of life lost due to violence.

A second perspective focuses on incident and socio-familial stressors. Research has demonstrated that the overwhelming majority of potential years of life due to homicide among African Americans are firearm-related [8]. Studies have also demonstrated that there is a strong relationship between race and ethnicity and family structure, whereas persons of color are more likely to be unmarried as adults and to reside in single-parent households as youth [33, 34]. In turn, these factors have been linked to racial disparities in interpersonal [25] and self-directed [35] violence. We therefore examine whether statistically accounting for incident characteristics (e,g., use of a firearm) and family characteristics (e.g., marital status and familial conflict) reduce racial and ethnic disparities in potential years of life lost due to violence.

A third view focuses on racial and ethnic differences in exposure to detrimental neighborhood conditions. Structural theorists speculate that the root causes of homicide are similar for all racial and ethnic groups, and that observed differences in violence across racial and ethnic groups are artifacts of the “divergent social worlds” in which non-Hispanic White persons and persons of color live [6]. Relatedly, researchers have argued that the external social world can contribute to an understanding of suicide [36]. Persons of color are more likely to reside in disadvantaged neighborhoods characterized by violence, a lack of access to community social services, a deficiency in community social support and informal social control, and increased contact with the police [34, 37]. Additionally, the multiple disadvantage model proposed by Lo and colleagues [12, 13] links racial and ethnic stratification across neighborhoods to individual, family, and incident characteristics that contribute to disparities in potential years of life lost due to violence. For example, contextual factors such as low socioeconomic status, high crime rates, and limited job opportunities—to which youth of color are disproportionately exposed—are associated with youth substance use, gang involvement, gun carrying, and exposure to violence, leaving youth of color particularly vulnerable to homicide victimization and suicidal behavior. The implication is that empirically accounting for these salient neighborhood characteristics may partly account for racial and ethnic differences in potential years of life lost due to violence.

### Current study

In summary, the literature indicates that homicide rates are higher among persons of color. While the literature has historically viewed suicide as a middle age, White, male phenomenon, there is evidence that rates of suicide among people of color—and especially young people of color—are increasing at higher rates than suicide among White persons. A more complete understanding of racial and ethnic disparities in homicide and suicide, however, requires a consideration of age at time of death. Accordingly, this study measures potential years of life lost to capture the number of years a person would have lived had they not died by homicide or suicide.

Using data from the National Violent Death Reporting System (NVDRS) and the American Community Survey (ACS), this study first establishes baseline estimates for potential years of life lost among Hispanic, non-Hispanic African American, non-Hispanic Asian or Pacific Islander, non-Hispanic American Indian or Alaska Native, and non-Hispanic White homicide victims, suicide decedents, and homicide-suicide perpetrators. Multilevel models then examine the extent to which racial and ethnic disparities in potential years of life lost are statistically accounted for by individual differences, incident and socio-familial factors, and contextual characteristics. The data provide a unique opportunity to examine a wide array of correlates of potential years of life lost among a racially and ethnically diverse sample of homicide victims, suicide decedents, and homicide-suicide perpetrators in the U.S.

## Materials and methods

### Data and sample

The National Violent Death Reporting System (NVDRS) is a state-based active web surveillance system of all persons who die by suicide, homicide, unintentional firearm fatality, legal intervention, and undetermined intent in the U.S. Established by the Centers for Disease Control and Prevention (CDC) in 2003, the NVDRS initially included data from seven states before becoming nationally representative in 2020.

The study sample includes information from 44 states and the District of Columbia. However, most records are from 17 states (see S1 Table), several states only reported a portion of incidents for some years (including California, Illinois, Pennsylvania, and Washington), and two states omitted data for some years due to incomplete case reporting (Hawaii and New York). As a result, the NVDRS currently represents only a sample of all violent deaths in the United States during the study time period.

The NVDRS is a pooled, cross-sectional time series with victims nested within census-designated places and states by year of death. Census-designated places are designed to provide information on settled concentrations of the population that are identifiable by name to locals, yet may or may not be legally incorporated. Census places are akin to cities, defined in coordination with local or tribal officials. Identified by Federal Information Processing Standard (FIPS) 55–3 codes, U.S. places includes all cities, county subdivisions (e.g., townships and census county divisions), American Indian and Alaska Native areas, several kinds of facilities (e.g., national parks, military installations, Coast Guard bases, and major airports), and counties and statistically equivalent legal and statistical entities as recognized by the U.S. Census Bureau [38]. Census places are always within a state, but may cross county and county subdivision lines.

The NVDRS integrates information from multiple sources to ensure data integrity. Required primary sources include death certificates (DC), coroner/medical examiner (CME) records, and law enforcement (LE) reports. When available, secondary data sources include crime lab and toxicology reports, hospital discharge data, court records, Child Fatality Review reports, Bureau of Alcohol, Tobacco, Firearms and Explosives (ATF) firearms trace data, Supplementary Homicide Reports (SHR), and National Incident-Based Reporting System records (NIBRS). While data collection and abstraction is conducted by each state, the CDC protects against systematic data errors by: using automated software validation during data entry; ensuring interrater reliability by requiring multiple coders to blindly recode data from the original source materials for a sample of cases; producing annual quality assurance reports; and providing coding support through training, email, and regular conference calls. The CDC completely anonymized the data prior to researcher access. The NVDRS is currently the most comprehensive and detailed data source on homicide and suicide in the U.S.

Contextual information derived from the U.S. Census Bureau’s American Community Survey (ACS) was appended to the NVDRS using census place geographic identifiers. First implemented in 2005, the ACS is a continuous, nationwide survey of households and group living quarters in the United States. With a response rate of approximately 95%, the ACS is currently the premier source of demographic, housing, and socioeconomic information in the U.S. The study midpoint (2009–2013) was used for estimation.

Our sample consists of all homicide victims, suicide decedents, and homicide-suicide perpetrators in the U.S. from 2003 to 2019 with valid geographic identifiers for census place of death. The final sample includes: 98,617 homicide victims nested within 93,629 incidents and 7,056 census-designated places; 230,527 suicide decedents nested within 13,408 places; and 3,962 homicide-suicide perpetrators nested in 2,057 places.

### Measures

#### Potential years of life lost

The outcome is potential years of life lost among homicide victims, suicide decedents, and homicide-suicide perpetrators. The NVDRS defines homicide as death resulting from the intentional use of force or power—threatened or actual—against another person, group, or community (ICD-10 codes X85-X99, U01-U03, Y00-Y09, and Y87.1). Homicide includes: incidents with intent to injure but not kill; deaths induced by the threat of force (e.g., heart attack); self-defense or “justifiable homicides” (not by a law enforcement officer); and intentional abuse or neglect. Suicide is defined as death resulting from the intentional use of force against oneself (ICD-10 codes X60-X84 and Y87.0). Homicide-suicide is defined as persons who committed suicide within 24 hours of fatally injuring another person [21]. Cases in which the cause of death was undetermined, accidental, or the result of legal intervention were excluded from the analysis.

**Fig 1:**
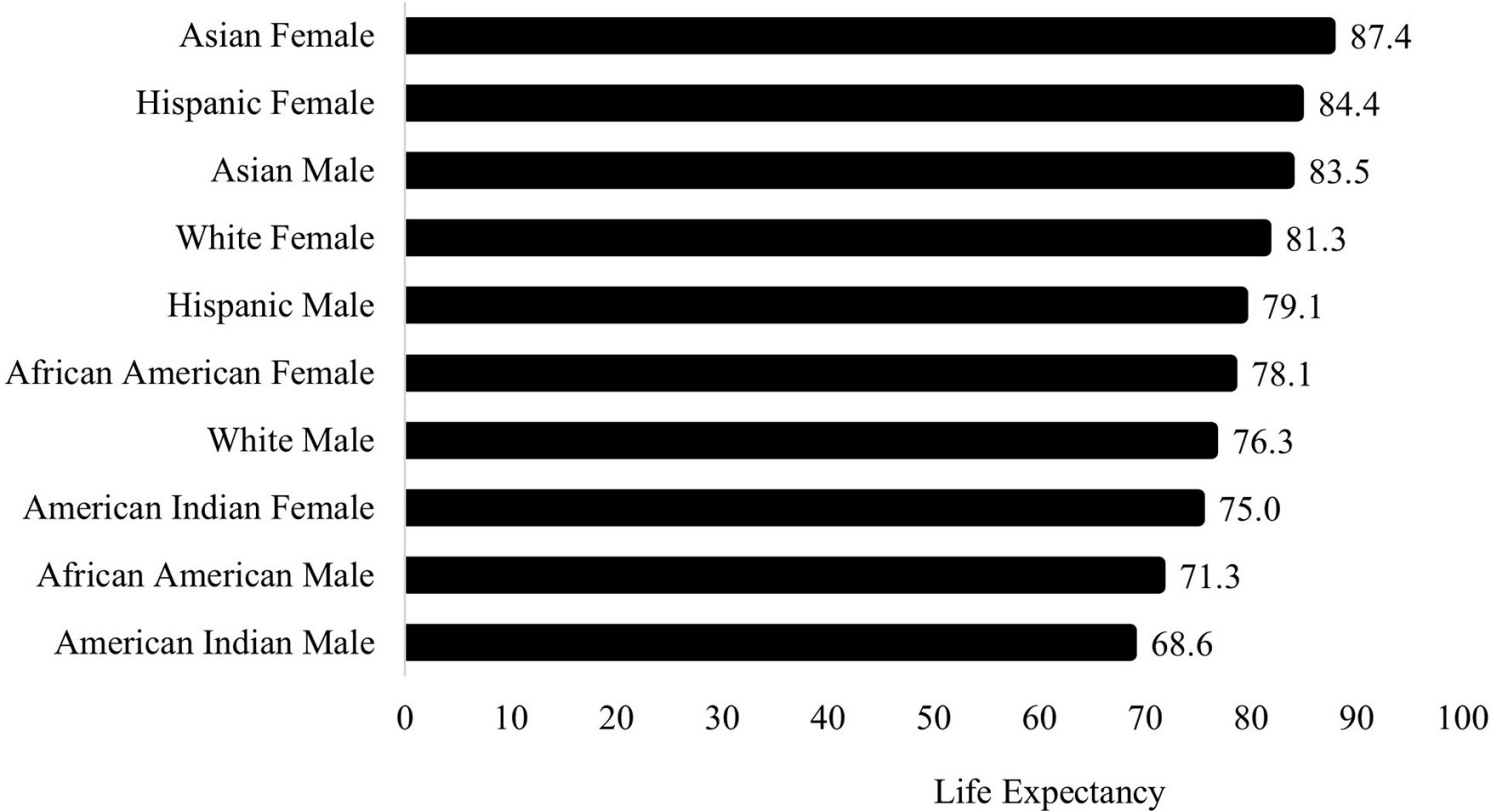
Life expectancy at birth, 2019, by race, ethnicity, and sex.

Previous research has calculated potential years of life lost by subtracting age at time of death from a standard life expectancy value of 75 [7, 39]. However, national health data is clear and consistent in demonstrating a strong correlation between race and ethnicity and life expectancy [40]. Similarly, there are differences in life expectancy across sex [40]. Fig 1 presents 2019 life expectancy values at birth in the U.S. by race, ethnicity, and sex; life expectancy values were obtained from the National Center for Health Statistics, National Vital Statistics System [40]. Given these documented disparities in life expectancy across race, ethnicity, and sex, we calculate potential years of life lost by subtracting age at time of death from a life expectancy value specific to an individual’s race, ethnicity, and sex. Consistent with established procedures [8, 41], if an individual is older than their life expectancy when they die, their potential years of life lost is coded zero (i.e., negative values are recoded zero). Effectively, only individuals who die prior to their life expectancy are included in the calculation [41].

Potential years of life lost measures the number of years a person would have lived if they had not died by a particular cause, in this study homicide or suicide [7]. Potential years of life lost is a useful tool for comparing the burden of death from homicide and suicide across groups; it highlights the disproportionate burden that premature deaths have on racial and ethnic groups [8]. Although there are many measures that could be used to quantify the relative importance of health outcomes such as mortality, this study focuses on potential years of life lost because of its specific emphasis on premature death. Crude mortality rates are dominated by health issues of the elderly without penalty for lengthy lifespan before death [8]. Morbidity measures like prevalence and incidence do not take into account when death occurs in a person’s lifespan [42]. Rates and risk estimates do not directly enumerate disparities in life expectancy or characterize the societal costs of homicide and suicide fatalities [9]. This study uses potential years of life lost in order to: measure premature death; characterize the societal costs of premature death among homicide victims, suicide decedents, and homicide-suicide perpetrators; give more weight to persons who died at younger ages; and focus on disparities in premature deaths across racial and ethnic groups [43]. Descriptive statistics of all study variables by homicide, suicide, and homicide-suicide are displayed in Table 1.

**Table 1:** Descriptive statistics for homicide victims (*N* = 98,617 persons, 93,629 incidents, 7,056 places), suicide decedents (*N* = 230,527 persons, 13,408 places), and homicide-suicide perpetrators (*N* = 3,962 persons, 2,057 places).

	Homicide Victims			Suicide Decedents			Homicide-Suicide Perpetrators		
Variable	% / Mean	N / (SD)	[Range]	% / Mean	N / (SD)	[Range]	% / Mean	N / (SD)	[Range]
Potential Years of Life Lost	41.52	(16.08)	[0–75]	29.46	(17.23)	[0–69]	29.54	(15.52)	[0–64]
Individual Differences									
Race and Ethnicity									
White	28.51%	28,115		82.64%	190,505		63.74%	2,525	
Hispanic	12.54%	12,370		6.01%	13,864		10.76%	426	
African American	54.43%	53,675		6.60%	15,211		20.26%	803	
Asian	1.41%	1,386		2.22%	5,119		3.23%	128	
American Indian	1.66%	1,633		1.34%	3,086		.98%	39	
Other	1.45%	1,438		1.19%	2,742		1.03%	41	
Sex									
Female	21.29%	20,994		22.52%	51,920		7.57%	300	
Male	78.71%	77,623		77.48%	178,607		92.43%	3,662	
Employment Status									
Unemployed	23.02%	22,707		15.35%	35,390		9.14%	362	
Low Job	53.10%	52,351		40.58%	93,553		46.39%	1,838	
Medium Job	17.06%	16,828		24.09%	55,532		26.95%	1,068	
High Job	6.82%	6,731		19.98%	46,052		17.52%	694	
Educational Attainment									
Less than High School	36.55%	36,041		17.88%	41,228		20.54%	814	
High School	44.22%	43,612		39.69%	91,485		44.54%	1,764	
Some College	15.21%	15,004		25.84%	59,576		22.68%	899	
College or Higher	4.02%	3,960		16.59%	38,238		12.24%	485	
Alcohol Problems	34.36%	33,886		34.34%	79,166		34.15%	1,353	
Drug Problems	45.40%	44,769		31.50%	72,621		26.18%	1,037	
Mental Health Problems	3.74%	3,693		49.21%	113,452		17.90%	709	
Married	---	---		32.67%	75,304		34.53%	1,368	
Incident Method^a^									
Shoot	71.04%	70,055		49.23%	113,486		89.74%	3,555	
Stab / Cut	12.43%	12,260		2.02%	4,647		2.64%	105	
Strangle / Asphyxiation	3.23%	3,183		27.72%	63,905		3.33%	132	
Bludgeon	5.68%	5,605		---	---		---	---	
Beat	4.24%	4,183		---	---		---	---	
Poison	---	---		15.29%	35,244		1.72%	68	
Other	3.38%	3,331		5.74%	13,245		2.57%	102	
Incident Location									
Home	49.45%	48,774		74.40%	171,528		75.68%	2,998	
Street	27.83%	27,441		4.48%	10,328		6.57%	260	
Car	8.24%	8,126		4.66%	10,737		5.92%	235	
Business	6.81%	6,714		3.80%	8,750		4.22%	167	
Other	7.67%	7,562		12.66%	29,184		7.61%	302	
Homicide Characteristics									
Offender Race and Ethnicity									
White	28.96%	28,563		---	---		---	---	
Hispanic	14.26%	14,064		---	---		---	---	
African American	53.56%	52,817		---	---		---	---	
Other	3.22%	3,173		---	---		---	---	
Offender Sex									
Female	10.61%	10,461		---	---		---	---	
Male	89.39%	88,156		---	---		---	---	
Victim-Offender Relationship									
Family	14.49%	14,285		---	---		---	---	
Friend	30.05%	29,644		---	---		---	---	
Romantic Partner	12.81%	12,635		---	---		---	---	
Acquaintance	27.58%	27,195		---	---		---	---	
Stranger	15.07%	14,858		---	---		---	---	
Homicide Type									
Homicide-Suicide	4.85%	4,783		---	---		---	---	
Multiple Homicide Offenders	20.80%	20,511		---	---		---	---	
Number of Victims									
Single Homicide	90.86%	89,609		---	---		---	---	
Double Homicide	6.87%	6,774		---	---		---	---	
Triple Homicide	1.44%	1,416		---	---		---	---	
Mass Murder	.83%	818		---	---		---	---	
Situational Characteristics									
Intimate Partner Violence	15.07%	14,864		---	---		---	---	
Argument	38.72%	38,187		---	---		---	---	
Gang Involvement	7.96%	7,854		---	---		---	---	
Victim Weapon	5.79%	5,714		---	---		---	---	
Drug Involvement	13.31%	13,129		---	---		---	---	
Criminal Involvement	30.76%	30,338		---	---		---	---	
Suicide and Homicide-Suicide Characteristics									
Suicide History									
History of Suicide Attempt	---	---		20.36%	46,924		3.84%	152	
Disclosed Suicide Intent	---	---		26.19%	60,372		11.63%	461	
Recent Exposure to Suicide	---	---		2.27%	5,223		.48%	19	
Recent Exposure to Death	---	---		6.38%	14,707		7.47%	296	
Stressors									
Intimate Partner Problems	---	---		27.87%	64,237		73.09%	2,896	
Family Problems	---	---		7.72%	17,797		9.01%	357	
Relationship Problems	---	---		4.63%	10,682		8.13%	322	
Criminal Problems	---	---		8.79%	20,256		19.79%	784	
Health Problems	---	---		21.75%	50,139		7.78%	308	
Job Problems	---	---		11.02%	25,411		5.43%	215	
School Problems	---	---		1.49%	3,426		.20%	8	
Money Problems	---	---		9.93%	22,894		7.20%	285	
Dual Suicide	---	---		.23%	526		.20%	8	
Place Characteristics									
Concentrated Disadvantage	.00	1.00	[-5.87–4.54]	.00	1.00	[-6.72–4.24]	.00	1.00	[-3.89–3.27]
Residential Stability	.00	1.00	[-5.65–2.54]	.00	1.00	[-5.71–2.23]	.00	1.00	[-4.47–2.76]
Racial and Ethnic Heterogeneity	.00	1.00	[-1.63–2.34]	.00	1.00	[-1.31–2.76]	.00	1.00	[-1.86–2.07]
Population	.00	1.00	[-3.68–4.35]	.00	1.00	[-3.63–4.83]	.00	1.00	[-3.16–3.83]

Abbreviation: SD = standard deviation.

^a^Represents suicide method for homicide-suicide.

#### Individual differences

The analysis includes several individual differences for homicide victims, suicide decedents, and homicide-suicide perpetrators. Demographic factors include biological sex (0 = male, 1 = female) and race and ethnicity: Hispanic, non-Hispanic African-American, non-Hispanic Asian or Pacific Islander, non-Hispanic American Indian or Alaska Native, non-Hispanic White, and other (includes two or more races).

Employment status was divided into four groups based on the type and skill level of work. The NVDRS provides Standard Occupational Classification (SOC) codes, which were matched to job zones provided by the U.S. Department of Labor/Employment and Training Administration’s Occupational Information Network (O*NET). Low status jobs include zones 1 and 2, or occupations that require a high school degree or less, minimal prior experience or job-related skill, and under one year of training (e.g., food preparation workers, rental clerks, security guards). Medium status jobs include zone 3, or occupations that require vocational school training or an associate’s degree, and multiple years of training (e.g., electricians, medical assistants, court reporters). High status jobs include zones 4 and 5, or occupations that typically require a four-year bachelor’s or graduate degree, and extensive training or experience (e.g., database administrators, graphic designers, lawyers, medical doctors). Victims who were retired, homemakers, or otherwise not working at the time of death were coded as unemployed.

Educational attainment was measured by four categories: less than high school; high school degree or equivalent; some college; and undergraduate college degree or higher (e.g., master’s or doctoral degree). Three binary (0 = no, 1 = yes) measures assessed whether the homicide victim, suicide decedent, or homicide-suicide perpetrator: had alcohol in their system at time of death; had amphetamines, opiates, marijuana, or cocaine in their system at time of death; or was currently suffering from or had a history of mental health issues. Marital status (1 = married, 0 = unmarried) was assessed for suicide decedents and homicide-suicide perpetrators.

#### Incident method and location

Method of death for homicide victims, suicide decedents, and homicide-suicide perpetrators included: shooting; stabbing or cutting with a sharp object; strangling or asphyxiation; bludgeoning with a blunt object (for homicide victims only); beating with personal weapons (for homicide victims only); poisoning (for suicide decedents and homicide-suicide perpetrators only); and other. Incident location was classified as: private residence or home; street or parking lot; car or other vehicle; business (e.g., hotel, bar, restaurant); and other.

#### Homicide characteristics

As the NVDRS is a dataset focused on persons who died, minimal information was available on homicide offenders. Nonetheless, the analysis includes available demographic information on homicide offenders, the nature of the victim-offender relationship, and homicide type. Demographic information on homicide offenders includes race and ethnicity and biological sex (0 = male, 1 = female). For homicide cases with multiple offenders (20.80%, *N* = 20,511), demographic information was aggregated across offenders as follows: offender sex was coded as female if at least one offender was female; offender race and ethnicity was coded as all Hispanic, all non-Hispanic African-American, all non-Hispanic White, and other.

Victim-offender relationship was coded into five mutually exclusive categories: family, friend, romantic partner, acquaintance, and stranger. For cases with multiple victim-offender relationships, the closest relationship was retained.

Three variables captured the type of homicide incident: whether the homicide perpetrator committed suicide following the homicide (0 = no, 1 = yes); whether the incident involved multiple offenders; and whether the incident was a single, double, triple, or mass (defined as four or more victims) homicide. A series of binary variables captured situational characteristics relevant to homicide, including whether (0 = no, 1 = yes) the incident: was the product of intimate partner violence; followed an argument or physical fight; was related to gang warfare; was precipitated by victim weapon use; was related to drug dealing, trading, or usage; and occurred during the commission of another crime.

#### Suicide and homicide-suicide characteristics

A series of binary measures (0 = no, 1 = yes) assessed the suicide decedent’s or homicide-suicide perpetrator’s experiences with suicide and death and socio-psychological stressors. Experiences with suicide and death included whether the decedent had previously attempted suicide, disclosed their intention to commit suicide to another person, had recently been exposed to a suicide of a family member or close friend, or had recently been exposed to the death of a family member or close friend. Stressors included whether the incident involved: problems with intimate partners, other family members, or other friends and acquaintances; criminal legal problems such as recent or pending arrest or evading law enforcement); physical health issues such as terminal disease, debilitating condition, or chronic pain; job problems such as unemployment, demotion, or increased pressure; school problems such as poor grades, bullying, or detention or suspension; and financial problems such as bankruptcy, debt, or foreclosure. The analysis also included a measure of dual suicide: whether or not the incident involved two or more concurrent suicides (e.g., a suicide pact).

#### Place characteristics

Contextual information at the place level was appended to incidents of lethal violence. Concentrated disadvantage is the weighted factor score of six indicators, including: the percentage of the population aged 18–64 living below the poverty line; percentage of the civilian workforce unemployed; percentage single female-headed household with children; percentage of the population aged 25 and older with a high school degree; percentage of the population aged 15 and older who were married; and median household income (last three items reverse coded).

Racial and ethnic heterogeneity was measured using Blau’s (1977) [44] index, calculated by summing the squared proportion of the population in each racial and ethnic group and then subtracting this sum from 1. This equation creates a variable ranging from 0 to 1 that takes into account both the relative sizes of the groups and the number of groups in the population, with higher values reflecting greater levels of heterogeneity.

Residential stability is the average of the proportion of the population living in owner-occupied housing and the proportion of residents living in the same house for the past year [45]. The natural log of the total population was included in models to control for city size.

### Analytic strategy

A series of three-level hierarchical linear regression models predict potential years of life lost among homicide victims in the U.S. from 2003 to 2019. Because some homicide victims were involved in double, triple, or mass (i.e., four or more victims) murder, the models nest individual victims within incidents and U.S. places. A series of two-level hierarchical linear regression models predict potential years of life lost among suicide decedents and homicide-suicide perpetrators. The models nest individuals within U.S. places.

Baseline models establish racial and ethnic differences in potential years of life lost. Three subsequent models add individual, incident, and contextual characteristics to examine the extent to which racial and ethnic disparities in potential years of life lost may be accounted for by these constellations of factors. Individual differences (sex, employment status, educational attainment, alcohol and drug problems, mental health problems) are added first. The next model includes incident method and location and situational characteristics of the incident. The final models add the neighborhood characteristics. Continuous variables are centered or standardized in the models. All variance inflation factors (VIF) are below 3.

## Results

**Fig 2:**
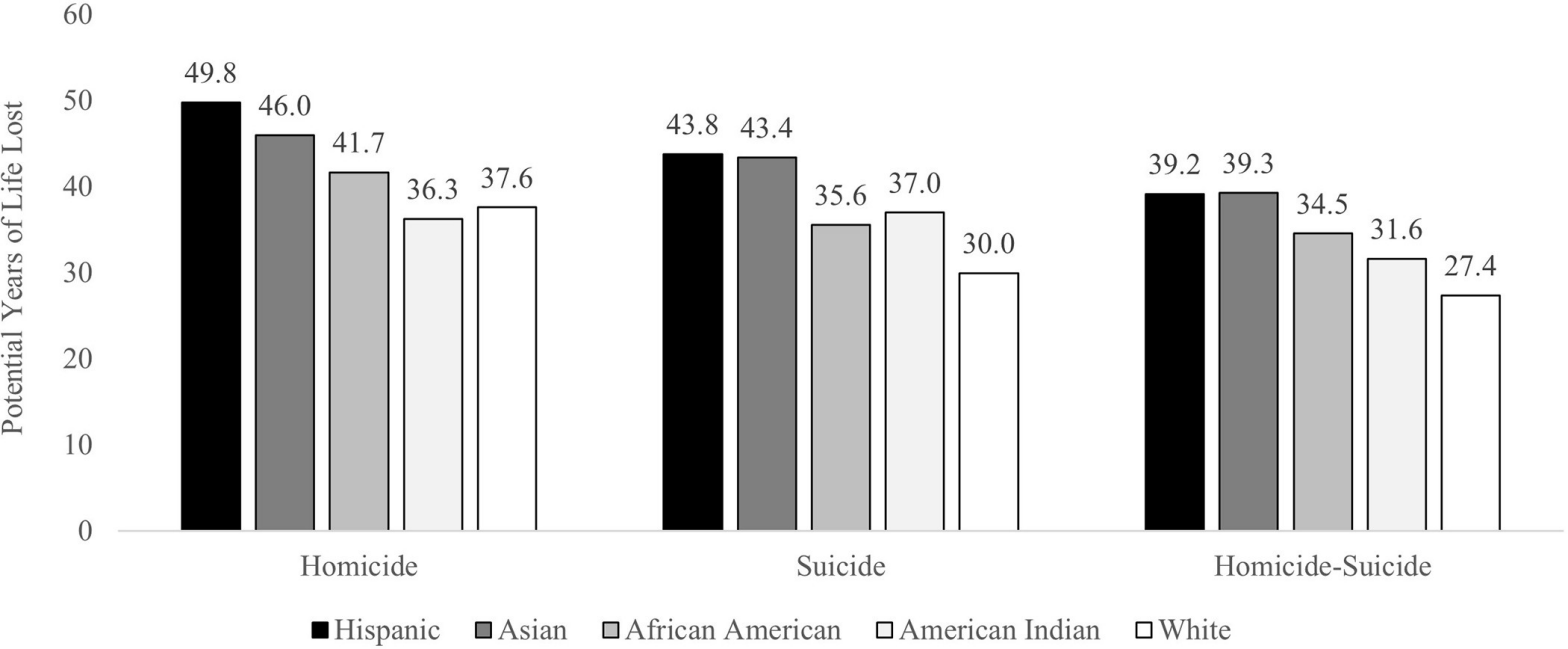
Average potential years of life lost among homicide victims, suicide decedents, and homicide-suicide perpetrators, by race and ethnicity.

Fig 2 presents the average potential years of life lost for homicide victims, suicide decedents, and homicide-suicide perpetrators, by race and ethnicity. Disparities are particularly apparent between White individuals and persons of color. White homicide victims had 37.6 potential years of life lost, on average, less than all other racial and ethnic groups except for American Indian homicide victims. Similarly, White suicide decedents had 30.0 potential years of life lost, on average, while White homicide-suicide perpetrators had 27.4 potential years of life lost, on average, less than all other racial and ethnic groups. Conversely, Hispanic individuals generally had the highest average potential years of life lost for all outcomes, followed by Asian, African American, and American Indian individuals, respectively. Note that all differences in average potential years of life lost across racial and ethnic groups are significant at *p* < .001, except for the difference across Hispanic and Asian persons for suicide (*ns*), African American and American Indian persons for homicide-suicide (*ns*), and Hispanic and Asian persons for homicide-suicide (*ns*). Results predicting potential years of life lost among homicide victims, suicide decedents, and homicide-suicide perpetrators are presented below.

### Homicide

Table 2 presents the hierarchical linear regression models predicting potential years of life lost among homicide victims. Model 1 provides baseline estimates for racial and ethnic disparities in potential years of life lost. Consistent with Fig 1, the results indicate that Hispanic, African American, and Asian homicide victims had 12.40, 4.14, and 8.61 more potential years of life lost, respectively, than White homicide victims. Potential years of life lost were statistically indistinguishable between Asian and White homicide victims.

**Table 2:** Hierarchical linear regression models predicting potential years of life lost among homicide victims (*N* = 98,617 persons, 93,629 incidents, 7,056 places).

	Model 1		Model 2		Model 3		Model 4	
Variable	Coef.	SE	Coef.	SE	Coef.	SE	Coef.	SE
Individual Differences								
Race and Ethnicity^a^								
Hispanic	12.40***	.22	10.62***	.21	9.17***	.23	9.16***	.23
African American	4.14***	.18	3.10***	.16	1.35***	.18	1.42***	.18
Asian	8.61***	.57	8.16***	.51	7.24***	.51	7.12***	.48
American Indian	-.97	.50	-1.74***	.46	-2.49***	.47	-1.96***	.50
Female			3.72***	.16	5.07***	.20	5.06***	.20
Employment Status^b^								
Unemployed			15.25***	.32	14.65***	.33	14.67***	.33
Low Job			6.66***	.24	6.33***	.24	6.37***	.24
Medium Job			4.15***	.26	3.92***	.26	3.95***	.26
Educational Attainment^c^								
Less than High School			6.51***	.18	6.30***	.18	6.36***	.18
High School			1.59***	.16	1.39***	.16	1.42***	.15
Alcohol Problems			-2.24***	.13	-2.05***	.13	-2.04***	.13
Drug Problems			.70**	.23	.29	.22	.25	.22
Mental Health Problems			-7.17***	.40	-6.69***	.38	-6.77***	.39
Incident Method^e^								
Stab					-4.90***	.20	-4.97***	.20
Strangle					-1.73***	.39	-1.82***	.39
Bludgeon					-5.77***	.33	-5.79***	.33
Beat					-2.61***	.45	-2.70***	.45
Incident Location^f^								
Street					1.67***	.14	1.63***	.14
Car					2.73***	.17	2.72***	.17
Business					.63**	.20	.61**	.20
Offender Characteristics								
Race and Ethnicity^a^								
Hispanic					1.56***	.26	1.56***	.26
African American					1.19***	.16	1.21***	.16
Female					1.18***	.20	1.19***	.20
Victim-Offender Relationship^d^								
Family					.80**	.25	.83***	.25
Friend					.05	.14	.07	.14
Romantic Partner					-.90*	.36	-.86*	.36
Acquaintance					.62***	.16	.64***	.16
Homicide Type								
Homicide-Suicide					-2.84***	.32	-2.95***	.32
Multiple Offenders					.74***	.15	.73***	.15
Number of Victims								
Double Homicide					.30	.22	.30	.22
Triple Homicide					3.36***	.56	3.36***	.56
Mass Murder					3.15**	1.01	3.14**	1.01
Situational Characteristics								
Intimate Partner Violence					.19	.24	.20	.24
Argument					-.23	.13	-.23	.12
Gang Involvement					2.66***	.23	2.60***	.22
Victim Weapon					1.26***	.18	1.26***	.18
Drug Involvement					.89***	.15	.86***	.15
Criminal Involvement					-2.19***	.15	-2.17***	.15
Place Characteristics								
Concentrated Disadvantage							.80***	.12
Residential Stability							-.57***	.13
Racial and Ethnic Heterogeneity							-.49***	.12
Population							.73***	.11
Variance Components^g^								
*e*	130.08		112.35		110.73		110.74	
*r*_*0*_	105.93		83.21		76.82		76.96	
*u*_*00*_	15.81		11.87		12.80		10.86	

Abbreviation: Coef.–coefficient; SE = standard error.

Notes: The models controls for 16 dummy variables representing year of death and “other” categories for race and ethnicity, incident method, and incident location.

^a^Reference = White

^b^Reference = Higher Job

^c^Reference = Some College or Higher

^d^Reference = Stranger

^e^Reference = Shoot

^f^Reference = Home

^g^The unconditional model (controlling for year of death) produced level 1 (*e*), level 2 (*r*_*0*_), and level 3 (*u*_*00*_) variance components of 130.97, 115.67, and 23.92, respectively.

*p < .05

**p < .01

***p < .001 (two-tailed tests).

Model 2 introduces the homicide victims’ individual differences to examine the personal factors that account for racial and ethnic disparities in potential years of life lost. Female homicide victims had more potential years of life lost than male homicide victims (β = 3.72, SE = .16). Unemployed victims (β = 15.25, SE = .32), victims with a low status job (β = 6.66, SE = .24), and victims with a medium status job (β = 4.15, SE = .26) had more potential years of life lost than victims with a highly prestigious job. Victims with less than a high school education (β = 6.51, SE = .18) or with a high school degree (β = 1.59, SE = .16) had more potential years of life lost than victims with at least some college education. Homicide victims with alcohol problems (β = -2.24, SE = .13) and mental health problems (β = -7.17, SE = .40) had fewer potential years of life lost than their counterparts, while homicide victims with drug problems (β = .70, SE = .23) had more potential years of life lost than their counterparts.

Controlling for these individual differences reduced the coefficient describing disparities in potential years of life lost between Hispanic and White homicide victims by 14.35% (from 12.40 to 10.62), the coefficient describing disparities in potential years of life lost between African American and White homicide victims by 25.12% (from 4.14 to 3.10), and the coefficient describing the gap in potential years of life lost between Asian and White homicide victims by 5.23% (from 8.61 to 8.16). These findings imply that individual differences among homicide victims are partly responsible for the increased potential years of life lost among Hispanic, African American, and Asian homicide victims, compared to White homicide victims. Also note that inclusion of the individual differences explained 13.63%, 21.45%, and 24.92% of the individual-, incident-, and place-level variation in potential years of life lost, respectively.

Model 3 adds the incident variables, homicide offender factors, and situational characteristics. Regarding homicide method and location, there were more potential years of life lost among victims of firearm homicide than among victims of stabbing (β = -4.90, SE = .20), strangling (β = -1.73, SE = .39), bludgeoning (β = -5.77, SE = .33), or beating (β = -2.61, SE = .45). Homicides occurring in the street (β = 1.67, SE = .14), in a car (β = 2.73, SE = .17), or in a business (β = .63, SE = .20) were associated with more potential years of life lost than homicides occurring in the home.

With respect to offender characteristics, victims of non-White offenders had more potential years of life lost than victims of White offenders (β = 1.56, SE = .26 for Hispanic offenders; β = 1.19, SE = .16 for African American offenders); and victims of female offenders had more potential years of life lost than victims of male offenders (β = 1.18, SE = .20). Relative to victims who were strangers, family victims (β = .80, SE = .25) and acquaintances (β = .62, SE = .16) had more potential years of life lost, and romantic partner victims had fewer potential years of life lost (β = -.90, SE = .36).

Victims of homicide-suicide had fewer potential years of life lost (β = -2.84, SE = .32), while the number of homicide offenders was positively associated with potential years of life lost (β = .74, SE = .15). The number of homicide victims involved in the incident was also relevant to potential years of life lost by the victim: victims of triple (β = 3.36, SE = .56) and mass (β = 3.15, SE = 1.01) murders had approximately three more potential years of life lost than victims of a single-victim homicide. With respect to the situational characteristics, homicides involving gangs (β = 2.66, SE = .23), victims who used weapons (β = 1.26, SE = .18), and drugs (β = .89, SE = .15) had more potential years of life lost, while homicides involving crime had fewer potential years of life lost (β = -2.19, SE = .15).

Controlling for these incident variables, homicide offender factors, and situational characteristics further reduced the coefficient describing disparities in potential years of life lost between Hispanic and White homicide victims by 13.65% (from 10.62 to 9.17), the coefficient describing disparities in potential years of life lost between African American and White homicide victims by 56.45% (from 3.10 to 1.35), and the coefficient describing disparities in potential years of life lost between Asian and White homicide victims by 11.27% (from 8.16 to 7.24). These findings imply that incident variables, homicide offender factors, and situational characteristics partly account for the increased potential years of life lost among Hispanic, African American, and Asian homicide victims compared to White homicide victims. In addition, the individual- and incident-level variance components were further reduced with the inclusion of these characteristics.

Model 4 adds the place-level characteristics. Victims had more years of life lost when they resided in more populous places (β = .73, SE = .11), in places with higher levels of concentrated disadvantage (β = .80, SE = .12), and in places with lower levels of residential stability (β = -.57, SE = .13) and racial and ethnic heterogeneity (β = -.49, SE = .11). Controlling for these place-level characteristics did not further reduce the coefficients describing racial and ethnic disparities in potential years of life lost, suggesting that the place-level factors included in the analysis did not account for the increased potential years of life lost among non-White relative to White homicide victims.

Overall, the inclusion of the study covariates reduced the coefficient describing disparities in potential years of life lost between Hispanic and White homicide victims by 26.13%, the coefficient describing disparities in potential years of life lost between African American and White homicide victims by 65.70%, and the coefficient describing disparities in potential years of life lost between Asian and White homicide victims by 17.31%. Additionally, the study covariates explained 14.87%, 27.35%, and 31.31% of the individual-, incident-, and place-level variation in potential years of life lost, respectively.

### Suicide

Table 3 presents the hierarchical linear regression models predicting potential years of life lost among suicide decedents. Model 1 provides baseline estimates for racial and ethnic disparities in potential years of life lost. The results indicate that Hispanic, African American, Asian, and American Indian suicide decedents had 14.37, 5.69, 13.87, and 6.48 more potential years of life lost, respectively, than White suicide decedents.

**Table 3:** Hierarchical linear regression models predicting potential years of life lost among suicide decedents (N = 230,527 persons, 13,408 places).

	Model 1		Model 2		Model 3		Model 4	
Variable	Coef.	SE	Coef.	SE	Coef.	SE	Coef.	SE
Individual Differences								
Race and Ethnicity^a^								
Hispanic	14.37***	.21	12.16***	.21	9.12***	.16	9.13***	.16
African American	5.69***	.17	3.93***	.15	1.90***	.13	1.92***	.13
Asian	13.87***	.30	13.11***	.27	10.80***	.25	10.79***	.25
American Indian	6.48***	.35	1.83***	.33	-.21	.30	-.05	.31
Female			3.22***	.09	4.18***	.08	4.19***	.08
Employment Status^b^								
Unemployed			15.10***	.16	12.26***	.14	12.26***	.15
Low Job			5.70***	.13	4.46***	.11	4.46***	.11
Medium Job			3.03***	.12	2.48***	.10	2.48***	.10
Educational Attainment^c^								
Less than High School			2.86***	.20	2.16***	.18	2.19***	.18
High School			.68***	.16	.69***	.13	.70***	.14
Some College			2.43***	.13	2.12***	.11	2.13***	.11
Alcohol Problems			3.68***	.09	1.57***	.08	1.56***	.08
Drug Problems			1.99***	.10	2.78***	.10	2.77***	.10
Mental Health Problems			1.57***	.08	1.06***	.07	1.05***	.07
Married			-7.37***	.07	-6.93***	.07	-6.92***	.07
Incident Method^d^								
Cut					-2.44***	.21	-2.47***	.21
Asphyxiation					3.79***	.09	3.78***	.09
Poison					-1.35***	.11	-1.36***	.11
Incident Location^e^								
Street					3.25***	.16	3.24***	.16
Car					3.25***	.14	3.25***	.14
Business					1.54***	.16	1.52***	.16
Suicide History								
History of Suicide Attempt					1.79***	.08	1.79***	.08
Disclosed Suicide Intent					.23***	.07	.23***	.07
Recent Exposure to Suicide					1.95***	.19	1.95***	.19
Recent Exposure to Death					-5.18***	.14	-5.18***	.14
Stressors								
Intimate Partner Problems					6.20***	.07	6.20***	.07
Family Problems					1.98***	.12	1.98***	.12
Relationship Problems					2.86***	.15	2.86***	.15
Criminal Problems					1.83***	.10	1.83***	.10
Health Problems					-12.14***	.08	-12.14***	.08
Job Problems					3.13***	.09	3.13***	.09
School Problems					14.71***	.21	14.69***	.21
Money Problems					-2.10***	.10	-2.11***	.10
Dual Suicide					-2.16*	.84	-2.17**	.84
Place Characteristics								
Concentrated Disadvantage							.22**	.07
Residential Stability							-.36***	.08
Racial and Ethnic Heterogeneity							-.12*	.05
Population							.01	.05
Variance Components^f^								
*e*	285.12		235.00		179.95		179.96	
*u*_*0*_	9.37		7.04		4.33		4.23	

Abbreviation: Coef.–coefficient; SE = standard error.

Notes: The models controls for 16 dummy variables representing year of death and “other” categories for race and ethnicity, incident method, and incident location.

^a^Reference = White

^b^Reference = Higher Job

^c^Reference = College or Higher

^d^Reference = Shoot

^e^Reference = Home

^f^The unconditional model produced level 1 (*e*) and level 2 (*u*_*0*_) variance components of 300.24 and 10.72, respectively.

*p < .05

**p < .01

***p < .001 (two-tailed tests).

Model 2 introduces the suicide decedents’ individual differences to examine the personal factors that may account for racial and ethnic disparities in potential years of life lost. Female suicide decedents had more potential years of life lost than their male counterparts (β = 3.22, SE = .09). Unemployed suicide decedents (β = 15.10, SE = .16), those with a low status job (β = 5.70, SE = .13), and those with a medium status job (β = 3.03, SE = .12) had more potential years of life lost than suicide decedents with a highly prestigious job. Suicide decedents with less than a high school education (β = 2.86, SE = .20), a high school education (β = .68, SE = .16), and some college (β = 2.43, SE = .13) had more potential years of life lost than those with a college degree. Additionally, suicide decedents with alcohol problems (β = 3.68, SE = .09), drug problems (β = 1.99, SE = .10), and mental health problems (β = 1.57, SE = .08) had more potential years of life lost than their counterparts. Married suicide decedents had fewer potential years of life lost than unmarried suicide decedents (β = -7.37, SE = .07).

Controlling for these individual differences reduced the coefficient describing disparities in potential years of life lost between Hispanic and White suicide decedents by 15.38% (from 14.37 to 12.16), the coefficient describing disparities in potential years of life lost between African American and White suicide decedents by 30.93% (from 5.69 to 1.90), and the coefficient describing disparities in potential years of life lost between American Indian and White suicide decedents by 71.76% (from 6.48 to 1.83). The coefficient describing the gap between Asian and White suicide decedents decreased marginally, by 5.48% (from 13.87 to 13.11). These findings imply that individual differences among suicide decedents partly account for the increased potential years of life lost among non-White relative to White suicide decedents. Additionally, the inclusion of the individual differences explained 17.58% and 24.87% of the individual- and place-level variation in potential years of life lost, respectively.

Model 3 adds the incident variables, suicide history, and stressors. Regarding suicide method, use of a firearm was associated with fewer potential years of life lost than asphyxiation (β = 3.79, SE = .09), but more potential years of life lost than cutting (β = -2.44, SE = .21) and poisoning (β = -1.35, SE = .11). Suicide occurring outside of the home was associated with more potential years of life lost than suicide occurring in the home (β = 3.25, SE = .16 in the street; β = 3.25, SE = .14 in the car; β = 1.54, SE = .16 in a business). Suicide history (β = 1.79, SE = .08), disclosed suicide attempt (β = .23, SE = .07), and recent exposure to suicide (β = 1.95, SE = .19) were positively associated with potential years of life lost, while recent exposure to death was negatively associated with potential years of life lost (β = -5.18, SE = .14). Suicide decedents with intimate partner problems (β = 6.20, SE = .07), family problems (β = 1.98, SE = .12), relationship problems (β = 2.86, SE = .15), criminal problems (β = 1.83, SE = .10), job problems (β = 3.13, SE = .09), and school problems (β = 14.71, SE = .21) had more potential years of life lost, while decedents with health problems (β = -12.14, SE = .08) and money problems (β = -2.10, SE = .10) had fewer potential years of life lost. Individuals who committed suicide with at least one other person had fewer potential years of life lost (β = -2.16, SE = .84).

Controlling for these incident variables, suicide history, and stressors further reduced the coefficient describing disparities in potential years of life lost between Hispanic and White suicide decedents by 25.00%, the coefficient for disparities in potential years of life lost between African American and White suicide decedents by 51.65%, and the coefficient for disparities between Asian and White suicide decedents by 17.62%. The coefficient describing the difference between American Indian and White suicide decedents was reduced to non-significance. These findings imply that incident variables, suicide history, and stressors partly account for the increased potential years of life lost among Hispanic, African American, and Asian suicide decedents compared to White suicide decedents. These factors wholly account for the elevated potential years of life lost among American Indian suicide decedents, relative to White suicide decedents. In addition, the individual-level variance component was reduced by 23.43% and the place-level variance component was reduced by 38.49% with the inclusion of these characteristics.

Model 4 adds the place-level characteristics. Suicide decedents had more potential years of life lost when they resided in places with higher levels of concentrated disadvantage (β = .22, SE = .07), and lower levels of residential stability (β = -.36, SE = .08) and racial and ethnic heterogeneity (β = -.12, SE = .05). Controlling for these place-level characteristics did not further reduce the coefficients describing racial and ethnic disparities in potential years of life lost, suggesting that the place-level factors included in the analysis do not account for the increased potential years of life lost among non-White relative to White suicide decedents.

Overall, the inclusion of the study covariates reduced the coefficients describing disparities in potential years of life lost between Hispanic, African American, Asian, American Indian, and White suicide decedents by 36.46%, 66.26%, 22.21%, and 100%, respectively. Additionally, the study covariates explained 36.88% and 54.86% of the individual- and place-level variation in potential years of life lost.

### Homicide-suicide

Table 4 presents the hierarchical linear regression models predicting potential years of life lost among homicide-suicide perpetrators. Model 1 indicates that Hispanic, African American, and Asian homicide-suicide perpetrators had 11.90, 7.17, and 11.96 more potential years of life lost, respectively, than White homicide-suicide perpetrators. Potential years of life lost were statistically indistinguishable between American Indian and White homicide-suicide perpetrators.

**Table 4:** Hierarchical linear regression models predicting potential years of life lost among homicide-suicide perpetrators (*N* = 3,962 persons, 2,057 places).

	Model 1		Model 2		Model 3		Model 4	
Variable	Coef.	SE	Coef.	SE	Coef.	SE	Coef.	SE
Individual Differences								
Race and Ethnicity^a^								
Hispanic	11.90***	.71	10.71***	.69	8.98***	.67	8.93***	.69
African American	7.17***	.54	5.91***	.52	4.21***	.51	4.14***	.55
Asian	11.96***	1.35	12.37***	1.28	11.34***	1.21	11.21***	1.22
American Indian	4.20	2.37	.67	1.91	.01	1.70	-.12	1.70
Female			5.20***	.79	5.60***	.74	5.62***	.74
Employment Status^c^								
Unemployed			12.72***	1.12	11.47***	1.08	11.40***	1.09
Low Job			5.50***	.81	4.52***	.75	4.51***	.75
Medium Job			3.66***	.82	2.89***	.78	2.86***	.78
Educational Attainment^b^								
Less than High School			1.26	1.00	1.28	.89	1.29	.90
High School			1.33	.96	1.54	.86	1.57	.86
Some College			2.61**	.91	2.74***	.83	2.75**	.83
Alcohol Problems			1.89***	.53	1.54**	.50	1.56**	.50
Drug Problems			2.95***	.55	2.73***	.55	2.74**	.55
Mental Health Problems			1.98**	.64	2.16***	.63	2.21***	.64
Married			-6.69***	.49	-5.94***	.46	-5.95***	.46
Incident Method^d^^,^^e^								
Cut					1.30	1.32	1.28	1.33
Asphyxiation					1.29	1.10	1.30	1.10
Poison					-1.27	1.69	-1.28	1.69
Incident Location^f^								
Street					4.69***	.81	4.68***	.81
Car					5.20***	.90	5.22***	.90
Business					1.82	1.02	1.75	1.02
Suicide History								
History of Suicide Attempt					2.36*	1.09	2.34*	1.09
Disclosed Suicide Intent					.44	.63	.47	.63
Recent Exposure to Suicide					.66	3.31	.71	3.31
Recent Exposure to Death					-.96	.82	-.94	.81
Stressors								
Intimate Partner Problems					1.86***	.54	1.84***	.54
Family Problems					1.66*	.76	1.67	.76
Relationship Problems					.61	.81	.59	.81
Criminal Problems					2.86***	.55	2.83***	.55
Health Problems					-14.31***	.79	-14.30***	.79
Job Problems					3.12***	.91	3.09***	.92
School Problems					14.50***	4.34	14.62***	4.38
Money Problems					-3.73***	.86	-3.71***	.86
Dual Suicide					9.70	6.15	9.81	6.20
Place Characteristics								
Concentrated Disadvantage							.23	.32
Residential Stability							-.53	.33
Racial and Ethnic Heterogeneity							.12	.29
Population							-.16	.25
Variance Components^g^								
*e*	216.37		185.21		163.37		163.24	
*u*_*0*_	5.12		5.52		4.93		5.09	

Abbreviation: Coef.–coefficient; SE = standard error.

Notes: The models controls for 16 dummy variables representing year of death and “other” categories for race and ethnicity, incident method, and incident location.

^a^Reference = White

^b^Reference = Higher Job

^c^Reference = College or Higher

^d^Reference = Shoot

^e^Represents suicide method

^f^Reference = Home

^g^The unconditional model produced level 1 (*e*) and level 2 (*u*_*0*_) variance components of 233.33 and 8.33, respectively.

*p < .05

**p < .01

***p < .001 (two-tailed tests).

Model 2 introduces the individual differences. Female homicide-suicide perpetrators had more potential years of life lost than male homicide-suicide perpetrators (β = 5.20, SE = .79). Unemployed homicide-suicide perpetrators (β = 12.72, SE = 1.12), those with a low status job (β = 5.50, SE = .81), and those with a medium status job (β = 3.66, SE = .82) had more potential years of life lost than homicide-suicide perpetrators with a highly prestigious job. Homicide-suicide perpetrators with some college education had more potential years of life lost than perpetrators with at least a college degree (β = 2.61, SE = .91). Homicide-suicide perpetrators with alcohol problems (β = 1.89, SE = .53), drug problems (β = 2.95, SE = .55), and mental health problems (β = 1.98, SE = .64) had more potential years of life lost than their counterparts. Married homicide-suicide perpetrators had fewer potential years of life lost than unmarried perpetrators (β = -6.69, SE = .49).

Controlling for these individual differences reduced the coefficient describing disparities in potential years of life lost between Hispanic and White homicide-suicide perpetrators by 10.00% (from 11.90 to 10.71) and the coefficient describing disparities in potential years of life lost between African American and White homicide-suicide perpetrators by 17.57% (from 7.17 to 5.91). These findings imply that individual differences among homicide-suicide perpetrators partly account for the increased potential years of life lost among Hispanic and African American homicide-suicide perpetrators compared to White perpetrators. The coefficient describing disparities in potential years of life lost between Asian and White homicide-suicide perpetrators was not reduced with inclusion of the individual differences. Also note that inclusion of the individual differences explained 14.40% of the individual-level variation in potential years of life lost.

Model 3 adds the incident variables, suicide history, and stressors. While suicide method was not significantly associated with potential years of life lost among homicide-suicide perpetrators, incident location was: homicide-suicides occurring in the street (β = 4.69, SE = .81) or in a car (β = 5.20, SE = .90) were associated with more potential years of life lost than those occurring in the home. Homicide-perpetrators with a history of suicide attempt had more potential years of life lost than their counterparts (β = 2.36, SE = 1.09). Homicide-suicide perpetrators with intimate partner problems (β = 1.86, SE = .54), family problems (β = 1.66, SE = .76), criminal problems (β = 2.86, SE = .55), job problems (β = 3.12, SE = .91), and school problems (β = 14.50, SE = 4.34) had more potential years of life lost, while perpetrators with health problems (β = -14.31, SE = .79) and money problems (β = -3.73, SE = .86) had fewer potential years of life lost.

Controlling for these incident variables, suicide history, and stressors further reduced the coefficient describing disparities in potential years of life lost between Hispanic and White homicide-suicide perpetrators by 16.15% (from 10.71 to 8.98) and the coefficient describing disparities in potential years of life lost between African American and White homicide-suicide perpetrators by 28.76% (from 5.91 to 4.21). The coefficient describing disparities in potential years of life lost between Asian and White perpetrators was reduced by 8.33% (from 12.37 to 11.34). These findings imply that incident variables, suicide history, and stressors partly account for the increased potential years of life lost among Hispanic, African American, and Asian homicide-suicide perpetrators compared to White perpetrators. In addition, the individual-level variance component was further reduced by 11.79% with the inclusion of these characteristics.

Model 4 adds the place-level characteristics. These factors were not significantly associated with potential years of life lost for homicide-suicide perpetrators, and controlling for these factors did not further reduce the coefficients describing racial and ethnic disparities in potential years of life lost.

Overall, the inclusion of the study covariates reduced the coefficient describing disparities in potential years of life lost between Hispanic and White homicide-suicide perpetrators by 24.96%, the coefficient describing disparities in potential years of life lost between African American and White homicide-suicide perpetrators by 42.26%, and the coefficient describing differences in potential years of life lost between Asian and White perpetrators by 6.27%. Additionally, the study covariates explained 24.56% of the individual-level variation in potential years of life lost.

### Supplemental analyses

Several supplemental analyses were performed to examine the robustness of the study findings. First, the measure of potential years of life lost was calculated by subtracting age at time of death from a life expectancy value specific to an individual’s race, ethnicity, and sex; and individuals older than their life expectancy value at time of death were assigned a value of zero [7, 8, 41]. The measure of potential years of life lost was recalculated using a standard life expectancy value of 75, as well as different life expectancy values (e.g., 65, 70, and 80), and the statistical models were re-estimated. In all cases, the results were substantively unchanged.

Second, the theoretical framework was predicated on the premise that individual differences, incident characteristics, and contextual factors vary by race and ethnicity; these differences, in turn, differentially expose persons of color to conditions that induce violent death at younger ages. While inclusion of these factors in the multilevel models clearly attenuated the racial and ethnic disparities in potential years of life lost, we did not directly assess the first part of the theoretical premise. That is, we did not examine differences in the study covariates across race and ethnicity. Instead, S2–S4 Tables provide a descriptive comparison of the study covariates across Hispanic, non-Hispanic African American, non-Hispanic Asian or Pacific Islander, non-Hispanic American Indian or Alaska Native, and non-Hispanic White persons. The results of the descriptive comparison provide evidence that non-White persons in the study sample were differentially exposed to a wide array of risk factors for homicide, suicide, and homicide-suicide.

Third, employment is likely endogenous with age. As such, the large coefficient for unemployment may be difficult to interpret, given that a significant portion of youth in the study sample likely were not eligible for meaningful employment. Models were re-estimated after excluding the employment variables, and the substantive results were unaltered.

## Discussion

Research indicates that the burden of violent death in the U.S. is disproportionate across racial and ethnic groups. The homicide rate is significantly elevated among the non-White population. The suicide rate, while consistently higher among White persons than among persons of color, decreased among White persons between 2018 and 2021, but increased among all other racial and ethnic groups [1]. Moreover, recent studies examining race-specific rates of potential years of life lost—a summary measure of premature mortality—indicate that persons of color may die at younger ages than their counterparts, leading to increased trauma among surviving family members, friends, and communities [8].

The findings herein correspond with this research. At baseline, Hispanic, Asian, and African American homicide victims had, on average, 12.2, 8.4, and 4.1 more potential years of life lost due to violence, respectively, than White homicide victims. Similarly, Hispanic, Asian, African American, and American Indian persons who died by suicide had 13.8, 13.4, 5.6, and 7.0 more potential years of life lost, respectively, than White suicide decedents. Hispanic, Asian, African American, and American Indian homicide-suicide perpetrators had 11.8, 11.9, 7.1, and 4.2 more potential years of life lost, respectively, than White homicide-suicide perpetrators.

Hierarchical linear regression models confirmed these racial and ethnic disparities and revealed several factors that partly accounted for them. Most notably, the results indicated that a significant portion of the observed racial and ethnic gaps in potential years of life lost were accounted for by an array of individual, incident, and situational characteristics. Inclusion of the study covariates reduced the observed gaps in potential years of life lost between Hispanic and White homicide victims, suicide decedents, and homicide-suicide perpetrators by 26%, 36%, and 25%, respectively. The gaps in potential years of life lost between African American and White homicide victims, suicide decedents, and homicide-suicide perpetrators were reduced by 66%, 66%, and 42%, respectively. The gaps between Asian and White homicide victims, suicide decedents, and homicide-suicide perpetrators were reduced by 17%, 22%, and 6%, respectively. And the gap between American Indian and White suicide decedents was reduced by 100%.

These findings are consistent with the premise that differential exposure to violence-inducing conditions among persons of color impact racial and ethnic disparities in potential years of life lost due to violence. The race-related individual differences predicting potential years of life lost include educational attainment, employment status, marital status, substance use problems, and mental health problems. Race-related incident and situational factors predicting potential years of life lost include access to firearms, intimate partner problems, family problems, health problems, job problems, and money problems. The empirical observation that these race-related factors statistically accounted for a meaningful percentage of racial and ethnic disparities in potential years of life lost suggests that these disparities are not unalterable. Rather, similar to the arguments made by Wilson in his sweeping examination of the relationship between race, education, and employment [34], racial and ethnic disparities in violence are rooted in economic and social conditions and can therefore be changed. Consequently, interventions that facilitate family allowances and childcare, increased educational opportunities, and access to middle- and upper-class employment opportunities can presumably reduce racial and ethnic disparities in violence and potential years of life lost due to violence. Similarly, providing communities of color with the resources to address intimate partner violence and health problems can potentially reduce racial and ethnic disparities in violence and potential years of life lost due to violence.

Racial and ethnic disparities in potential years of life lost due to violence may also be rooted in larger systemic issues and institutionalized racism. For example, prior work in both criminology and public health discusses differences in culture as a potential source of racial and ethnic disparities in lethal violence victimization. Sampson and Wilson (1995 [46]) posit that extreme levels of social disorganization and concentrated disadvantage socially isolate communities of color from mainstream values and norms, promoting alternative cognitive landscapes. This “code of the street” mentality tolerates violence—even lethal violence—as a socially acceptable way to promote social status and prevent victimization [37]. In turn, this cultural adaptation may lead to racial and ethnic disparities in homicide victimization and age at violent death, as young men of color disproportionately live in communities that endorse street values [47–49].

While the “code of the street” typically references interpersonal violence, other cultural differences may shape racial and ethnic disparities in self-harm and suicide. Historical and contemporary mistreatment by the U.S. government and public health authorities (such as the 1932–1972 Tuskegee Syphilis Study and the appropriation of Henrietta Lacks’ cancer cell line) have fostered a general distrust of the medical community, including mental healthcare, for people of color [50]. African American and Hispanic persons are more likely than their White counterparts to believe mental illness is stigmatizing, is a sign of personal weakness, and can be resolved without medical treatment [51–53]. Similarly, the desire to “save face” and uphold the “model minority” stereotype discourage help-seeking behavior among Asian Americans [54, 55]. American Indians cite embarrassment and lack of belief in healthcare services as barrier to help-seeking behavior [56]. Such beliefs inhibit the utilization of mental health services and informal support from social networks.

Compounding these cultural effects are racial and ethnic disparities in access to and quality of emergency medical care and mental healthcare. Structural barriers to treatment include: distance to care and lack of transportation; financial issues and lack of insurance; difficulties communicating (language barriers); rarity of providers of color; and provider implicit bias and racism [57]. Implicit bias may be particularly influential as a source of racial and ethnic disparities in trauma and emergency department care, as the nature of the injury requires physicians to make decisions under pressure with limited information [58]. One meta-analysis found that the odds of mortality following trauma are 19% higher for African Americans relative to Whites [59]. Even further, some research suggests that African American adolescents who sustain firearm injuries receive lower triage acuity scores and are four times more likely to die than White juveniles [60, 61]. Similar issues plague the behavioral healthcare system—among individuals who attempted suicide, the odds of not receiving mental healthcare services were 3.20 times greater for African Americans and 1.36 times greater for Hispanic persons than for their White counterparts [62]. There is also a general failure in healthcare access, efficacy, and available resources (e.g., staff and infrastructure) for American Indians compared with other Americans [63]. Similarly, Asian Americans and Pacific Islanders are less likely than non-Hispanic Whites to have access to health care (e.g., a personal healthcare provider) and preventive services (e.g., colonoscopy, screening for cervical or prostate cancer, cholesterol check) [64]. Further, the fact that disparities in access to mental healthcare have increased for people of color following the COVID-19 global pandemic [65] has profound implications for future work on racial and ethnic disparities in age at violent death.

The current study’s inability to control for cultural factors, quality medical care, and access to mental healthcare may contribute to our inability to fully account for racial and ethnic disparities in potential years of life lost due to violence. We turn to colleagues to examine the racial and ethnic disparities in potential years of life lost that remain unexplained, given the likelihood that we excluded factors that are correlated with race and ethnicity and predictive of potential years of life lost due to violence.

The findings and our conclusions may be tempered by several data limitations. First, the majority of deaths in the study sample occurred in 17 states (S1 Table), which are not equally dispersed across the four regions of the country and tend to be more densely populated. The unbalanced data across region and urbanicity limits generalizability and introduces the potential for bias. As such, the estimates in this study may be artificially inflated or attenuated and should be interpreted with caution. Second, because the NVDRS is a dataset focused on persons who died in the U.S., we were unable to include comparison groups of persons who did not die by homicide or suicide. While beyond the scope of this study, including a comparison group of persons who did not die by homicide or suicide would allow for an examination of the factors that can increase the risk for violent death or protect against it. Existing data sources such as the Mortality Disparities in American Community Study (MDAC) would be helpful in this regard. Third, we recognize the importance of studying individuals who belong to two or more racial or ethnic groups, as well as racial and ethnic subgroups (e.g., Mexican and Puerto Rican Hispanics, and Chinese and Japanese Asians). Yet, given the relative rarity with which these populations are represented in the data, we restricted this study to the five principal racial and ethnic groups in the U.S.

## Conclusion

With these limitations in mind, we conclude by reiterating the key observation that violent deaths in the U.S. are disproportionate across racial and ethnic groups. Using a measure of potential years of life lost to more completely assess these racial and ethnic inequities, we found that Hispanic, African American, Asian or Pacific Islander, and American Indian or Alaska Native homicide victims, suicide decedents, and homicide-suicide perpetrators died significantly younger than their White counterparts. These disparities were partly accounted for by the inclusion of a broad array of individual, socio-familial, incident, and situational characteristics, suggesting that that they are rooted in family factors, interpersonal stressors, systemic bias, institutionalized racism, and cultural differences. As such, racial and ethnic differences in the causes of violence—and potential years of life lost due to violence—are amenable to change. Ultimately, addressing racial and ethnic disparities among homicide victims, suicide decedents, and homicide-suicide perpetrators requires a full understanding of their extent, etiology, and consequences. We encourage future research to continue to disaggregate violent deaths by race, ethnicity, age, and gender to foster this understanding.

## Supporting information

S1 Table Availability of data for the national violent death reporting system, by state and year. (DOCX)

S2 Table Descriptive statistics for homicide victims, by race and ethnicity. (DOCX)

S3 Table Descriptive statistics for suicide decedents, by race and ethnicity. (DOCX)

S4 Table Descriptive statistics for homicide-suicide perpetrators, by race and ethnicity. (DOCX)
