# Supplementary material for: Disproportionate burden of violence: Explaining racial and ethnic disparities in potential years of life lost among homicide victims, suicide decedents, and homicide-suicide perpetrators
Source: PLoS One. 2024 Feb 7;19(2):e0297346. doi: 10.1371/journal.pone.0297346 (PMC10849238; doi:10.1371/journal.pone.0297346)
Supplement: S2 Table — (DOCX) [file pone.0297346.s002.docx]

**S2 Table. Descriptive Statistics for Homicide Victims, by Race and Ethnicity.**

|  | **Hispanic (*n* = 12,370)** | | | **African American (*n* = 53,675)** | | | **Asian (*n* = 1,386)** | | | **American Indian (*n* = 1,633)** | | | **White (*n* = 28,115)** | | |
| --- | --- | --- | --- | --- | --- | --- | --- | --- | --- | --- | --- | --- | --- | --- | --- |
| **Variable** | **%/Mean** | **N/(SD)** | **[Range]** | **%/Mean** | **N/(SD)** | **[Range]** | **%/Mean** | **N/(SD)** | **[Range]** | **%/Mean** | **N/(SD)** | **[Range]** | **%/Mean** | **N/(SD)** | **[Range]** |
| Potential Years of Life Lost*** | 49.78 | (14.19) | [0–84.4] | 41.67 | (13.53) | [0–78.1] | 45.98 | (18.43) | [0–87.4] | 36.26 | (15.62) | [0–75] | 37.57 | (19.39) | [0–81.3] |
| Individual Differences |  |  |  |  |  |  |  |  |  |  |  |  |  |  |  |
| Sex*** |  |  |  |  |  |  |  |  |  |  |  |  |  |  |  |
| Female | 18.15% | 2,245 |  | 14.13% | 7,585 |  | 33.55% | 465 |  | 24.39% | 398 |  | 35.34% | 9,936 |  |
| Male | 81.85% | 10,125 |  | 85.87% | 46,090 |  | 66.45% | 921 |  | 75.61% | 1,235 |  | 64.66% | 18,179 |  |
| Employment Status*** |  |  |  |  |  |  |  |  |  |  |  |  |  |  |  |
| Unemployed | 23.04% | 2,851 |  | 23.75% | 12,745 |  | 21.63% | 300 |  | 21.95% | 359 |  | 21.46% | 6,035 |  |
| Low Job | 57.97% | 7,170 |  | 55.57% | 29,828 |  | 49.45% | 685 |  | 55.60% | 907 |  | 46.40% | 13,045 |  |
| Medium Job | 14.85% | 1,837 |  | 15.97% | 8,572 |  | 16.17% | 224 |  | 18.06% | 295 |  | 20.15% | 5,666 |  |
| High Job | 4.14% | 512 |  | 4.71% | 2,530 |  | 12.75% | 177 |  | 4.39% | 72 |  | 11.99% | 3,369 |  |
| Educational Attainment*** |  |  |  |  |  |  |  |  |  |  |  |  |  |  |  |
| Less than High School | 48.91% | 6,049 |  | 36.89% | 19,799 |  | 28.82% | 400 |  | 42.75% | 698 |  | 30.01% | 8,438 |  |
| High School | 38.04% | 4,706 |  | 46.85% | 25,152 |  | 38.47% | 533 |  | 40.78% | 666 |  | 42.74% | 12,018 |  |
| Some College | 11.12% | 1,376 |  | 14.15% | 7,594 |  | 17.11% | 237 |  | 14.87% | 243 |  | 19.04% | 5,352 |  |
| College or Higher | 1.93% | 239 |  | 2.11% | 1,130 |  | 15.60% | 216 |  | 1.60% | 26 |  | 8.21% | 2,307 |  |
| Alcohol Problems*** | 38.41% | 4,752 |  | 34.20% | 18,357 |  | 27.45% | 381 |  | 54.68% | 893 |  | 32.15% | 9,038 |  |
| Drug Problems*** | 46.97% | 5,811 |  | 48.58% | 26,075 |  | 31.13% | 432 |  | 37.84% | 618 |  | 40.23% | 11,312 |  |
| Mental Health Problems*** | 2.70% | 334 |  | 2.30% | 1,237 |  | 3.69% | 51 |  | 4.18% | 68 |  | 6.98% | 1,962 |  |
| Homicide Method*** |  |  |  |  |  |  |  |  |  |  |  |  |  |  |  |
| Shoot | 66.12% | 8,178 |  | 81.36% | 43,669 |  | 60.16% | 833 |  | 47.13% | 770 |  | 55.88% | 15,712 |  |
| Stab | 16.37% | 2,025 |  | 9.21% | 4,943 |  | 19.06% | 264 |  | 24.05% | 392 |  | 15.85% | 4,456 |  |
| Strangle | 3.14% | 389 |  | 1.82% | 979 |  | 5.31% | 74 |  | 3.50% | 57 |  | 5.72% | 1,609 |  |
| Bludgeon | 5.87% | 726 |  | 3.37% | 1,809 |  | 6.14% | 85 |  | 9.52% | 156 |  | 9.62% | 2,703 |  |
| Beat | 5.09% | 630 |  | 2.19% | 1,177 |  | 5.03% | 70 |  | 10.29% | 168 |  | 7.32% | 2,058 |  |
| Other | 3.41% | 422 |  | 2.05% | 1,098 |  | 4.30% | 60 |  | 5.51% | 90 |  | 5.61% | 1,577 |  |
| Homicide Location*** |  |  |  |  |  |  |  |  |  |  |  |  |  |  |  |
| Home | 44.70% | 5,531 |  | 41.91% | 22,498 |  | 48.64% | 674 |  | 57.85% | 945 |  | 65.15% | 18,314 |  |
| Street | 31.04% | 3,839 |  | 35.22% | 18,902 |  | 18.81% | 261 |  | 17.59% | 287 |  | 13.66% | 3,841 |  |
| Car | 8.89% | 1,098 |  | 9.93% | 5,332 |  | 7.34% | 102 |  | 5.25% | 86 |  | 5.01% | 1,410 |  |
| Business | 6.74% | 834 |  | 6.63% | 3,557 |  | 17.61% | 244 |  | 5.42% | 89 |  | 6.69% | 1,881 |  |
| Other | 8.63% | 1,068 |  | 6.31% | 3,386 |  | 7.60% | 105 |  | 13.89% | 226 |  | 9.49% | 2,669 |  |
| Offender Race/Ethnicity*** |  |  |  |  |  |  |  |  |  |  |  |  |  |  |  |
| White | 16.26% | 2,012 |  | 15.02% | 8,065 |  | 14.38% | 199 |  | 13.06% | 213 |  | 63.95% | 17,978 |  |
| African American | 36.06% | 4,461 |  | 73.36% | 39,368 |  | 33.46% | 464 |  | 19.42% | 317 |  | 27.54% | 7,742 |  |
| Hispanic | 43.73% | 5,408 |  | 10.40% | 5,585 |  | 22.09% | 306 |  | 28.74% | 469 |  | 5.98% | 1,683 |  |
| Other | 3.95% | 489 |  | 1.22% | 657 |  | 30.07% | 417 |  | 38.78% | 634 |  | 2.53% | 712 |  |
| Offender Sex |  |  |  |  |  |  |  |  |  |  |  |  |  |  |  |
| Female | 9.09% | 1,125 |  | 9.55% | 5,124 |  | 11.19% | 155 |  | 13.30% | 217 |  | 13.08% | 3,677 |  |
| Male | 90.91% | 11,245 |  | 90.45% | 48,551 |  | 88.81% | 1,231 |  | 86.70% | 1,416 |  | 86.92% | 24,438 |  |
| Victim-Offender Relationship*** |  |  |  |  |  |  |  |  |  |  |  |  |  |  |  |
| Family | 13.12% | 1,624 |  | 12.47% | 6,696 |  | 15.03% | 207 |  | 19.04% | 311 |  | 18.40% | 5,173 |  |
| Friend | 29.06% | 3,593 |  | 32.16% | 17,261 |  | 21.89% | 304 |  | 31.07% | 507 |  | 26.92% | 7,571 |  |
| Romantic Partner | 11.09% | 1,372 |  | 8.41% | 4,514 |  | 18.67% | 259 |  | 13.75% | 225 |  | 21.64% | 6,083 |  |
| Acquaintance | 28.83% | 3,567 |  | 31.18% | 16,736 |  | 21.98% | 305 |  | 24.20% | 395 |  | 20.77% | 5,839 |  |
| Stranger | 17.90% | 2,214 |  | 15.78% | 8,468 |  | 22.43% | 311 |  | 11.94% | 195 |  | 12.27% | 3,449 |  |
| Homicide Type |  |  |  |  |  |  |  |  |  |  |  |  |  |  |  |
| Homicide-Suicide*** | 4.18% | 517 |  | 1.48% | 793 |  | 11.74% | 163 |  | 3.74% | 61 |  | 11.31% | 3,180 |  |
| Multiple Homicide Offenders | 23.18% | 2,868 |  | 23.35% | 12,533 |  | 19.50% | 270 |  | 16.31% | 266 |  | 15.25% | 4,287 |  |
| Number of Victims |  |  |  |  |  |  |  |  |  |  |  |  |  |  |  |
| Single Homicide | 90.64% | 11,211 |  | 92.36% | 49,573 |  | 85.10% | 1,180 |  | 92.52% | 1,511 |  | 88.35% | 24,839 |  |
| Double Homicide | 6.83% | 845 |  | 6.15% | 3,302 |  | 9.58% | 132 |  | 5.82% | 95 |  | 8.20% | 2,306 |  |
| Triple Homicide | 1.61% | 200 |  | 1.09% | 583 |  | 3.50% | 49 |  | 1.29% | 21 |  | 1.91% | 536 |  |
| Mass Murder | .92% | 114 |  | .40% | 217 |  | 1.82% | 25 |  | .37% | 6 |  | 1.54% | 434 |  |
| Situational Characteristics |  |  |  |  |  |  |  |  |  |  |  |  |  |  |  |
| Intimate Partner Violence*** | 13.18% | 1,631 |  | 10.55% | 5,665 |  | 20.15% | 279 |  | 16.53% | 270 |  | 24.18% | 6,797 |  |
| Argument*** | 40.54% | 5,015 |  | 38.34% | 20,579 |  | 30.80% | 427 |  | 49.30% | 805 |  | 38.23% | 10,747 |  |
| Gang Involvement*** | 15.16% | 1,876 |  | 9.45% | 5,072 |  | 7.21% | 100 |  | 6.40% | 105 |  | 2.04% | 572 |  |
| Victim Weapon*** | 5.30% | 656 |  | 6.61% | 3,546 |  | 3.91% | 54 |  | 5.33% | 87 |  | 4.59% | 1,290 |  |
| Drug Involvement*** | 12.17% | 1,506 |  | 14.96% | 8,031 |  | 7.17% | 99 |  | 10.14% | 166 |  | 11.14% | 3,132 |  |
| Criminal Involvement*** | 27.82% | 3,441 |  | 31.45% | 16,881 |  | 39.31% | 545 |  | 24.38% | 398 |  | 30.73% | 8,639 |  |
| Place Characteristics |  |  |  |  |  |  |  |  |  |  |  |  |  |  |  |
| Concentrated Disadvantage*** | -.38 | .75 | [-4.05-2.61] | -.68 | .74 | [-3.60-3.30] | -.04 | .83 | [-3.40-3.49] | -.60 | 1.03 | [-5.20-1.85] | -.17 | .81 | [-5.87-4.54] |
| Residential Stability*** | -.85 | .75 | [-5.65-2.54] | -.98 | .69 | [-5.65-2.54] | -.78 | .79 | [-5.65-1.96] | -.46 | .97 | [-5.24-2.54] | -.53 | .87 | [-5.65-2.54] |
| Racial/Ethnic Heterogeneity*** | 1.26 | .70 | [-1.63-2.34] | 1.20 | .64 | [-1.63-2.34] | 1.23 | .71 | [-1.63-2.27] | .67 | 1.03 | [-1.63-2.20] | .59 | .95 | [-1.63-2.27] |
| Population*** | 1.98 | 1.18 | [-3.02-4.35] | 1.99 | 1.13 | [-2.57-4.35] | 1.89 | 1.06 | [-2.37-4.35] | .60 | 1.41 | [-3.08-3.91] | 1.10 | 1.25 | [-3.68-4.35] |

Abbreviation: SD = standard deviation.

**p* < .05; ***p* < .01; ****p* < .001 (two-tailed tests for differences across racial and ethnic groups).
