# Supplementary material for: Disproportionate burden of violence: Explaining racial and ethnic disparities in potential years of life lost among homicide victims, suicide decedents, and homicide-suicide perpetrators
Source: PLoS One. 2024 Feb 7;19(2):e0297346. doi: 10.1371/journal.pone.0297346 (PMC10849238; doi:10.1371/journal.pone.0297346)
Supplement: S3 Table — (DOCX) [file pone.0297346.s003.docx]

**S3 Table. Descriptive Statistics for Suicide Decedents, by Race and Ethnicity.**

|  | **Hispanic (*n* = 13,864)** | | | **African American (*n* = 15,211)** | | | **Asian (*n* = 5,119)** | | | **American Indian (*n* = 3,086)** | | | **White (*n* = 190,505)** | | |
| --- | --- | --- | --- | --- | --- | --- | --- | --- | --- | --- | --- | --- | --- | --- | --- |
| **Variable** | **%/Mean** | **N/(SD)** | **[Range]** | **%/Mean** | **N/(SD)** | **[Range]** | **%/Mean** | **N/(SD)** | **[Range]** | **%/Mean** | **N/(SD)** | **[Range]** | **%/Mean** | **N/(SD)** | **[Range]** |
| Potential Years of Life Lost*** | 43.78 | (15.84) | [0–74.4] | 35.56 | (15.79) | [0–70.1] | 43.38 | 18.28 | [0–76.4] | 36.98 | 14.06 | [0–66.0] | 29.96 | (17.34) | [0–75.3] |
| Individual Differences |  |  |  |  |  |  |  |  |  |  |  |  |  |  |  |
| Sex*** |  |  |  |  |  |  |  |  |  |  |  |  |  |  |  |
| Female | 19.98% | 2,770 |  | 20.05% | 3,050 |  | 32.30% | 1,654 |  | 24.14% | 745 |  | 22.60% | 43,045 |  |
| Male | 80.02% | 11,094 |  | 79.95% | 12,161 |  | 67.70% | 3,465 |  | 75.86% | 2,341 |  | 77.40% | 147,460 |  |
| Employment Status*** |  |  |  |  |  |  |  |  |  |  |  |  |  |  |  |
| Unemployed | 21.36% | 2,962 |  | 19.79% | 3,010 |  | 26.24% | 1,343 |  | 26.42% | 815 |  | 13.99% | 26,652 |  |
| Low Job | 48.67% | 6,747 |  | 46.57% | 7,084 |  | 32.73% | 1,675 |  | 50.21% | 1,549 |  | 39.55% | 75,330 |  |
| Medium Job | 19.79% | 2,743 |  | 21.12% | 3,213 |  | 19.37% | 992 |  | 16.55% | 511 |  | 24.92% | 47,483 |  |
| High Job | 10.18% | 1,412 |  | 12.52% | 1,904 |  | 21.66% | 1,109 |  | 6.82% | 211 |  | 21.54% | 41,040 |  |
| Educational Attainment*** |  |  |  |  |  |  |  |  |  |  |  |  |  |  |  |
| Less than High School | 32.98% | 4,572 |  | 22.91% | 3,485 |  | 17.65% | 903 |  | 34.73% | 1,072 |  | 16.04% | 30,561 |  |
| High School | 37.08% | 5,141 |  | 40.77% | 6,202 |  | 28.55% | 1,462 |  | 41.00% | 1,265 |  | 40.07% | 76,344 |  |
| Some College | 22.06% | 3,058 |  | 25.41% | 3,865 |  | 24.37% | 1,248 |  | 20.32% | 627 |  | 26.27% | 50,039 |  |
| College or Higher | 7.88% | 1,093 |  | 10.91% | 1,659 |  | 29.43% | 1,506 |  | 3.95% | 122 |  | 17.62% | 33,561 |  |
| Alcohol Problems*** | 37.12% | 5,147 |  | 32.01% | 4,870 |  | 28.06% | 1,436 |  | 40.24% | 1,242 |  | 34.42% | 65,563 |  |
| Drug Problems*** | 35.82% | 4,966 |  | 32.25% | 4,905 |  | 23.95% | 1,226 |  | 30.24% | 933 |  | 31.40% | 59,810 |  |
| Mental Health Problems*** | 43.38% | 6,014 |  | 41.69% | 6,342 |  | 45.73% | 2,341 |  | 37.30% | 1,151 |  | 50.57% | 96,344 |  |
| Married*** | 27.54% | 3,818 |  | 23.26% | 3,538 |  | 36.79% | 1,883 |  | 19.84% | 612 |  | 33.98% | 64,735 |  |
| Suicide Method*** |  |  |  |  |  |  |  |  |  |  |  |  |  |  |  |
| Shoot | 33.36% | 4,626 |  | 47.04% | 7,155 |  | 21.85% | 1,119 |  | 36.43% | 1,124 |  | 51.56% | 98,233 |  |
| Cut | 2.38% | 329 |  | 1.78% | 271 |  | 2.99% | 153 |  | 2.10% | 65 |  | 1.99% | 3,787 |  |
| Asphyxiation | 44.74% | 6,204 |  | 29.91% | 4,550 |  | 49.27% | 2,522 |  | 47.09% | 1,453 |  | 25.33% | 48,252 |  |
| Poison | 11.12% | 1,541 |  | 10.95% | 1,665 |  | 11.48% | 588 |  | 10.13% | 313 |  | 16.16% | 30,778 |  |
| Other | 8.40% | 1,164 |  | 10.32% | 1,570 |  | 14.41% | 737 |  | 4.25% | 131 |  | 4.96% | 9,455 |  |
| Suicide Location*** |  |  |  |  |  |  |  |  |  |  |  |  |  |  |  |
| Home | 71.61% | 9,928 |  | 67.90% | 10,329 |  | 69.53% | 3,560 |  | 71.44% | 2,203 |  | 75.34% | 143,523 |  |
| Street | 5.80% | 804 |  | 6.31% | 959 |  | 5.07% | 259 |  | 3.88% | 120 |  | 4.22% | 8,042 |  |
| Car | 4.11% | 570 |  | 5.98% | 910 |  | 5.08% | 260 |  | 2.91% | 90 |  | 4.62% | 8,794 |  |
| Business | 3.63% | 503 |  | 3.80% | 577 |  | 4.93% | 252 |  | 2.80% | 87 |  | 3.79% | 7,221 |  |
| Other | 14.85% | 2,059 |  | 16.01% | 2,436 |  | 15.39% | 788 |  | 18.97% | 586 |  | 12.03% | 22,925 |  |
| Suicide History |  |  |  |  |  |  |  |  |  |  |  |  |  |  |  |
| History of Suicide Attempt*** | 23.18% | 3,214 |  | 17.66% | 2,686 |  | 20.08% | 1,028 |  | 22.92% | 708 |  | 20.30% | 38,672 |  |
| Disclosed Suicide Intent*** | 27.71% | 3,841 |  | 23.45% | 3,567 |  | 21.89% | 1,121 |  | 30.38% | 938 |  | 26.28% | 50,062 |  |
| Recent Exposure to Suicide*** | 2.38% | 330 |  | 1.25% | 190 |  | 1.69% | 87 |  | 5.30% | 164 |  | 2.30% | 4,381 |  |
| Recent Exposure to Death*** | 5.42% | 751 |  | 5.33% | 811 |  | 4.14% | 212 |  | 7.34% | 227 |  | 6.58% | 12,539 |  |
| Stressors |  |  |  |  |  |  |  |  |  |  |  |  |  |  |  |
| Intimate Partner Problems*** | 36.15% | 5,013 |  | 28.37% | 4,316 |  | 22.83% | 1,169 |  | 37.40% | 1,154 |  | 27.10% | 51,623 |  |
| Family Problems*** | 10.25% | 1,421 |  | 7.15% | 1,088 |  | 8.45% | 433 |  | 11.17% | 345 |  | 7.48% | 14,244 |  |
| Relationship Problems*** | 5.70% | 790 |  | 4.20% | 639 |  | 3.93% | 201 |  | 6.81% | 210 |  | 4.53% | 8,623 |  |
| Criminal Problems*** | 11.18% | 1,551 |  | 11.69% | 1,779 |  | 6.45% | 330 |  | 13.89% | 429 |  | 8.33% | 15,876 |  |
| Health Problems*** | 12.03% | 1,668 |  | 11.70% | 1,779 |  | 14.83% | 759 |  | 10.69% | 330 |  | 23.72% | 45,193 |  |
| Job Problems*** | 9.50% | 1,317 |  | 9.70% | 1,475 |  | 11.33% | 580 |  | 6.42% | 198 |  | 11.31% | 21,545 |  |
| School Problems*** | 2.56% | 355 |  | 2.41% | 367 |  | 4.00% | 205 |  | 2.90% | 89 |  | 1.22% | 2,320 |  |
| Money Problems*** | 8.27% | 1,147 |  | 8.14% | 1,238 |  | 10.09% | 516 |  | 5.99% | 185 |  | 10.27% | 19,566 |  |
| Dual Suicide*** | .11% | 15 |  | .09% | 14 |  | .43% | 22 |  | .19% | 6 |  | .24% | 467 |  |
| Place Characteristics |  |  |  |  |  |  |  |  |  |  |  |  |  |  |  |
| Concentrated Disadvantage*** | -.30 | .78 | [-3.84-3.34] | -.56 | .86 | [-5.03-3.53] | .07 | .85 | [-3.70-3.27] | -.78 | 1.16 | [-6.71-2.51] | -.11 | .84 | [-6.17-4.24] |
| Residential Stability*** | -.82 | .84 | [-5.71-2.23] | -.98 | .84 | [-5.71-2.23] | -.82 | .94 | [-5.71-2.23] | -.49 | .95 | [-5.40-2.23] | -.59 | .90 | [-5.71-2.23] |
| Racial/Ethnic Heterogeneity*** | 1.36 | .83 | [-1.31-2.76] | 1.44 | .77 | [-1.31-2.74] | 1.45 | .88 | [-1.31-2.74] | .60 | 1.12 | [-1.31-2.64] | .73 | 1.00 | [-1.31-2.76] |
| Population*** | 2.08 | 1.27 | [-2.75-4.83] | 2.02 | 1.25 | [-2.72-4.83] | 2.14 | 1.23 | [-2.29-4.83] | .68 | 1.43 | [-3.63-4.83] | 1.36 | 1.21 | [-3.22-4.83] |

Abbreviation: SD = standard deviation.

**p* < .05; ***p* < .01; ****p* < .001 (two-tailed tests for differences across racial and ethnic groups).
