# Supplementary material for: Disproportionate burden of violence: Explaining racial and ethnic disparities in potential years of life lost among homicide victims, suicide decedents, and homicide-suicide perpetrators
Source: PLoS One. 2024 Feb 7;19(2):e0297346. doi: 10.1371/journal.pone.0297346 (PMC10849238; doi:10.1371/journal.pone.0297346)
Supplement: S4 Table — (DOCX) [file pone.0297346.s004.docx]

**S4 Table. Descriptive Statistics for Homicide-Suicide Perpetrators, by Race and Ethnicity.**

|  | **Hispanic (*n* = 426)** | | | **African American (*n* = 803)** | | | **Asian (*n* = 128)** | | | **American Indian (*n* = 39)** | | | **White (*n* = 2,525)** | | |
| --- | --- | --- | --- | --- | --- | --- | --- | --- | --- | --- | --- | --- | --- | --- | --- |
| **Variable** | **%/Mean** | **N/(SD)** | **[Range]** | **%/Mean** | **N/(SD)** | **[Range]** | **%/Mean** | **N/(SD)** | **[Range]** | **%/Mean** | **N/(SD)** | **[Range]** | **%/Mean** | **N/(SD)** | **[Range]** |
| Potential Years of Life Lost*** | 39.16 | (13.18) | [0–69.4] | 34.54 | (12.28) | [0–60.3] | 39.26 | 15.76 | [0–72.4] | 31.63 | 14.78 | [0–52.6] | 27.37 | (15.85) | [0–64.3] |
| Individual Differences |  |  |  |  |  |  |  |  |  |  |  |  |  |  |  |
| Sex*** |  |  |  |  |  |  |  |  |  |  |  |  |  |  |  |
| Female | 7.98% | 34 |  | 5.36% | 43 |  | 11.74% | 15 |  | 15.38% | 6 |  | 7.96% | 201 |  |
| Male | 92.02% | 392 |  | 94.64% | 760 |  | 88.26% | 113 |  | 84.62% | 33 |  | 92.04% | 2,324 |  |
| Employment Status*** |  |  |  |  |  |  |  |  |  |  |  |  |  |  |  |
| Unemployed | 9.55% | 41 |  | 9.67% | 78 |  | 13.30% | 17 |  | 22.82% | 9 |  | 8.50% | 215 |  |
| Low Job | 57.25% | 244 |  | 56.45% | 453 |  | 37.01% | 47 |  | 54.36% | 21 |  | 41.74% | 1,053 |  |
| Medium Job | 23.02% | 98 |  | 22.88% | 184 |  | 28.64% | 37 |  | 16.92% | 7 |  | 28.77% | 727 |  |
| High Job | 10.18% | 43 |  | 11.00% | 88 |  | 21.05% | 27 |  | 5.90% | 2 |  | 20.99% | 530 |  |
| Educational Attainment*** |  |  |  |  |  |  |  |  |  |  |  |  |  |  |  |
| Less than High School | 34.51% | 147 |  | 22.27% | 179 |  | 21.67% | 28 |  | 35.64% | 14 |  | 17.26% | 436 |  |
| High School | 38.98% | 166 |  | 47.82% | 383 |  | 40.77% | 51 |  | 45.90% | 18 |  | 44.51% | 1,124 |  |
| Some College | 18.44% | 79 |  | 21.99% | 177 |  | 13.69% | 18 |  | 13.08% | 5 |  | 24.33% | 614 |  |
| College or Higher | 8.07% | 34 |  | 7.92% | 64 |  | 23.87% | 31 |  | 5.38% | 2 |  | 13.90% | 351 |  |
| Alcohol Problems*** | 37.14% | 158 |  | 34.82% | 280 |  | 22.54% | 29 |  | 56.15% | 22 |  | 34.64% | 850 |  |
| Drug Problems*** | 27.92% | 119 |  | 25.85% | 208 |  | 21.36% | 27 |  | 23.59% | 9 |  | 26.18% | 661 |  |
| Mental Health Problems*** | 16.19% | 69 |  | 11.17% | 90 |  | 10.49% | 13 |  | 7.95% | 3 |  | 20.98% | 530 |  |
| Married*** | 31.72% | 135 |  | 25.62% | 206 |  | 39.51% | 51 |  | 23.85% | 9 |  | 37.92% | 958 |  |
| Suicide Method*** |  |  |  |  |  |  |  |  |  |  |  |  |  |  |  |
| Shoot | 82.88% | 353 |  | 92.43% | 741 |  | 81.07% | 104 |  | 78.46% | 31 |  | 90.72% | 2,291 |  |
| Cut | 5.30% | 23 |  | 1.46% | 12 |  | 6.34% | 8 |  | 7.95% | 3 |  | 2.27% | 57 |  |
| Asphyxiation | 7.32% | 31 |  | 2.80% | 23 |  | 7.82% | 10 |  | 5.38% | 2 |  | 2.50% | 63 |  |
| Poison | .70% | 3 |  | .87% | 7 |  | .00% | 0 |  | 2.56% | 1 |  | 2.26% | 57 |  |
| Other | 3.80% | 16 |  | 2.44% | 20 |  | 4.77% | 6 |  | 5.64% | 2 |  | 2.25% | 57 |  |
| Suicide Location*** |  |  |  |  |  |  |  |  |  |  |  |  |  |  |  |
| Home | 70.82% | 301 |  | 69.83% | 561 |  | 66.28% | 85 |  | 71.80% | 28 |  | 78.97% | 1,993 |  |
| Street | 9.81% | 42 |  | 9.28% | 75 |  | 5.40% | 7 |  | 10.26% | 4 |  | 5.05% | 128 |  |
| Car | 4.58% | 20 |  | 8.50% | 68 |  | 9.15% | 12 |  | 2.56% | 1 |  | 5.23% | 132 |  |
| Business | 6.95% | 30 |  | 4.88% | 39 |  | 6.57% | 8 |  | 5.13% | 2 |  | 3.48% | 88 |  |
| Other | 7.84% | 33 |  | 7.51% | 60 |  | 12.60% | 16 |  | 10.26% | 4 |  | 7.27% | 184 |  |
| Suicide History |  |  |  |  |  |  |  |  |  |  |  |  |  |  |  |
| History of Suicide Attempt*** | 4.55% | 19 |  | 1.82% | 15 |  | .00% | 0 |  | .00% | 0 |  | 4.65% | 117 |  |
| Disclosed Suicide Intent | 13.09% | 56 |  | 9.57% | 77 |  | 2.58% | 3 |  | 23.33% | 9 |  | 12.31% | 311 |  |
| Recent Exposure to Suicide | .23% | 1 |  | .30% | 2 |  | .78% | 1 |  | 2.56% | 1 |  | .50% | 13 |  |
| Recent Exposure to Death* | 5.02% | 21 |  | 5.66% | 45 |  | 7.20% | 9 |  | 5.64% | 2 |  | 8.58% | 217 |  |
| Stressors |  |  |  |  |  |  |  |  |  |  |  |  |  |  |  |
| Intimate Partner Problems*** | 79.56% | 339 |  | 79.53% | 639 |  | 67.84% | 87 |  | 65.90% | 26 |  | 70.24% | 1,774 |  |
| Family Problems*** | 8.35% | 36 |  | 4.83% | 39 |  | 6.57% | 8 |  | 10.51% | 4 |  | 10.57% | 267 |  |
| Relationship Problems | 7.51% | 32 |  | 6.60% | 53 |  | 6.49% | 8 |  | 7.69% | 3 |  | 8.90% | 225 |  |
| Criminal Problems* | 22.36% | 95 |  | 22.17% | 178 |  | 20.50% | 26 |  | 34.87% | 14 |  | 18.38% | 464 |  |
| Health Problems*** | 2.39% | 10 |  | 1.63% | 13 |  | 7.28% | 9 |  | 7.69% | 3 |  | 10.76% | 271 |  |
| Job Problems** | 3.36% | 14 |  | 3.74% | 30 |  | 5.79% | 7 |  | 5.13% | 2 |  | 6.32% | 160 |  |
| School Problems | .23% | 1 |  | .14% | 1 |  | .78% | 1 |  | .00% | 0 |  | .20% | 5 |  |
| Money Problems*** | 5.54% | 24 |  | 3.60% | 29 |  | 4.15% | 5 |  | 5.13% | 2 |  | 8.93% | 226 |  |
| Dual Suicide | .00% | 0 |  | .00% | 0 |  | .00% | 0 |  | .00% | 0 |  | .32% | 8 |  |
| Place Characteristics |  |  |  |  |  |  |  |  |  |  |  |  |  |  |  |
| Concentrated Disadvantage*** | -.31 | .76 | [-2.78-1.79] | -.59 | .88 | [-3.87-2.41] | .03 | .83 | [-1.56-2.35] | -.66 | .85 | [-2.61-.77] | -.14 | .85 | [-2.84-2.97] |
| Residential Stability*** | -.80 | .82 | [-4.67-1.76] | -.92 | .87 | [-3.67-1.90] | -.83 | .87 | [-3.34-1.56] | -.84 | .84 | [-2.58-.54] | -.55 | .90 | [-4.60-2.23] |
| Racial/Ethnic Heterogeneity*** | 1.37 | .82 | [-1.08-2.66] | 1.46 | .73 | [-1.31-2.66] | 1.41 | .89 | [-1.19-2.64] | .54 | .97 | [-1.26-2.33] | .69 | 1.00 | [-1.31-2.66] |
| Population*** | 1.96 | 1.23 | [-2.53-4.83] | 1.88 | 1.21 | [-1.64-4.83] | 2.00 | 1.01 | [-.19-4.83] | .38 | 1.17 | [-1.65-3.27] | 1.21 | 1.22 | [-2.46-4.83] |

Abbreviation: SD = standard deviation.

**p* < .05; ***p* < .01; ****p* < .001 (two-tailed tests for differences across racial and ethnic groups).
